# Altered Gut Microbiota Profiles in Sows and Neonatal Piglets Associated with Porcine Epidemic Diarrhea Virus Infection

**DOI:** 10.1038/s41598-017-17830-z

**Published:** 2017-12-12

**Authors:** Deping Song, Qi Peng, Yanjun Chen, Xinrong Zhou, Fanfan Zhang, Anqi Li, Dongyan Huang, Qiong Wu, Yu Ye, Houjun He, Leyi Wang, Yuxin Tang

**Affiliations:** 1 Key Laboratory for Animal Health of Jiangxi Province, Nanchang, Jiangxi 330045 China; 2 Department of Preventive Veterinary Medicine, College of Animal Science and Technology, Jiangxi Agricultural University, Nanchang, Jiangxi 330045 China; 3 Department of Veterinary Clinical Medicine and the Veterinary Diagnostic Laboratory, College of Veterinary Medicine, University of Illinois, Urbana, IL 61802 USA

## Abstract

Porcine epidemic diarrhea virus (PEDV) is a devastating cause of diarrhea in pigs worldwide. Most of studies have focused on molecular and pathogenic characterization of PEDV, whereas there were limited studies in understanding the role of gut microbiota (GM) in viral-associated diarrhea. Here, using the Illumina MiSeq platform, we examined and compared the impact of PEDV infection on the GM of sows and their piglets less than 10 days old. Our results showed that PEDV caused alternations in the structure and abundance of GM from levels of phylum to genus, and even species. For sows, a significant decrease of observed species was found in diarrheal sows than that in healthy sows (p < 0.05). The unweighted and weighted UniFrac distances also revealed considerable segregations of GM structure among healthy, asymptomatic, and diarrheal sows. For piglets, *Bacteroidetes*, the dominant bacteria in healthy piglets, were replaced by *Firmicutes* in asymptomatic and diarrheal piglets. The abundances of *Fusobacteria* and *Proteobacteria* were also remarkably increased in asymptomatic piglets and diarrheal piglets when compared to those of the healthy piglets. Our findings demonstrated that PEDV infection caused severe perturbations of GM, reduced probiotic bacteria, and enriched pathogenic bacteria.

## Introduction

Since 2010, the global swine industry has experienced outbreaks of severe diarrhea caused by variant porcine epidemic diarrhea virus (PEDV)^1–3^. The variant PEDV caused severe diarrhea, vomiting, dehydration, and high morbidity and mortality in newborn piglets as well as sporadic diarrhea in sows and finishing pigs in China in 2010. Afterwards, the outbreaks have been reported in Europe^4^, America^5,6^, and other Asian countries^7,8^. Pigs suffered from diarrheal syndromes were unresponsive to antibiotics treatment and common management practices, and were even not protected from prior immunization with attenuated and/or inactivated CV777-based PEDV vaccines^9,10^.

Most of previous studies were mainly focused on the characterization of PEDV, virus isolation, pathology, and molecular evolution. However, there were only limited studies about the effects of the mucosal microflora residents on health and their roles during PEDV infection. The mammalian gut is a complex ecological niche, containing bacteria, eukaryotes, viruses, and even archaeon^11^. It has been demonstrated that colonized gut microbiota (GM) are important for the health and development of animals via aiding colonic fermentation^12^, stimulating the immune system^13^, defensing pathogens^14^, and improving energy harvest^15^. Studies on humans unraveled that diarrhea^16^ and bowel inflammation^17^ would distort GM in normal gut. In pigs, a few studies that examined the GM of healthy pigs under different growth stages or breeds demonstrated *Bacteroidetes* and *Firmicutes* are predominant phyla regardless of ages and breeds, while dynamic changes in percentage of GM at both phylum and genus levels were observed under different growth stages and conditions including age, weaning and colitis^18–25^. Remarkable alternations of fecal microbiota were reported in neonatal piglets with diarrhea (NNPD) of unknown etiology^26,27^. It was reported genus *Enterococcus* was 24 times and phylum *Fusobacteria* was doubled in abundance in diarrheal piglets compared to that of the healthy piglets. Studies on diarrheal weaning pigs and newborn piglets with natural infection of PEDV have also demonstrated GM perturbations from phylum to genus levels^28,29^. Both aforementioned studies showed that *Fusobacteria* was higher in the PEDV infected pigs than in the healthy groups, and either *Verrucomicrobia* ^28^ or most commensal bacteria^29–31^ were found significantly lower in infected piglets due to the dysbiosis caused by PEDV. However, both studies just focused on the nursing or >10-day-old suckling piglets, but did not observe the GM variations in their mother sows and newborn piglets under 10 days old. Therefore, further investigations on GM perturbations in PEDV-infected sows and newborn piglets are indeed needed.

In the present study, we attempted to elucidate the GM profiles in naturally PEDV infected sows and healthy sows, and the offspring delivered by these sows. We firstly presented a systematically comparative evaluation on the microbiota profiles of healthy sows and their relevant offspring by using the universal primers covering 16 S rRNA (small subunit of the ribosome in bacteria) V1~V3 hypervariable regions and the Illumina MiSeq platform. We then analyzed the GM alterations in sows and <10-day-old newborn piglets with symptomless and diarrhea during variant PEDV infection.

## Results

### Sequencing consistency of mock community (MC)

To evaluate the primers 27 F and 534 R for 16 S rRNA V1~V3 hypervariable regions and analytic workflows for MiSeq next generation sequencing (NGS) platform, a MC was constructed by using 15 known bacterial species belonging to 13 genera, in three phyla (Table S1a). The equimolar pooled genomic DNAs of MC along with that of clinic samples were amplified and then sequenced by an Illumina MiSeq paired-end (PE) 300 run. After quality control, the reads were processed using Quantitative Insights Into Microbial Ecology (QIIME) v 1.9.0. At the phylum level, 99.27% reads were assigned to three phyla (*Firmicutes*, *Proteobacteria*, and *Actinobacteria*); 92.35% assigned reads hit the 13 genera of MC (Table S1b). The abundance of pooled bacteria determined by NGS showed an average of 85% agreement rate with that of qPCR (Detailed information was provided in Appendix 1. Experimental Procedures). Hence, it was proved that the primers 27 F and 534R-based PCR could accurately amplify the 16 S rDNA of the microflora and thus the methodology established was appropriate for unveiling the microbial communities in clinic samples.

### Sequencing data of clinical samples

**Figure 1:**
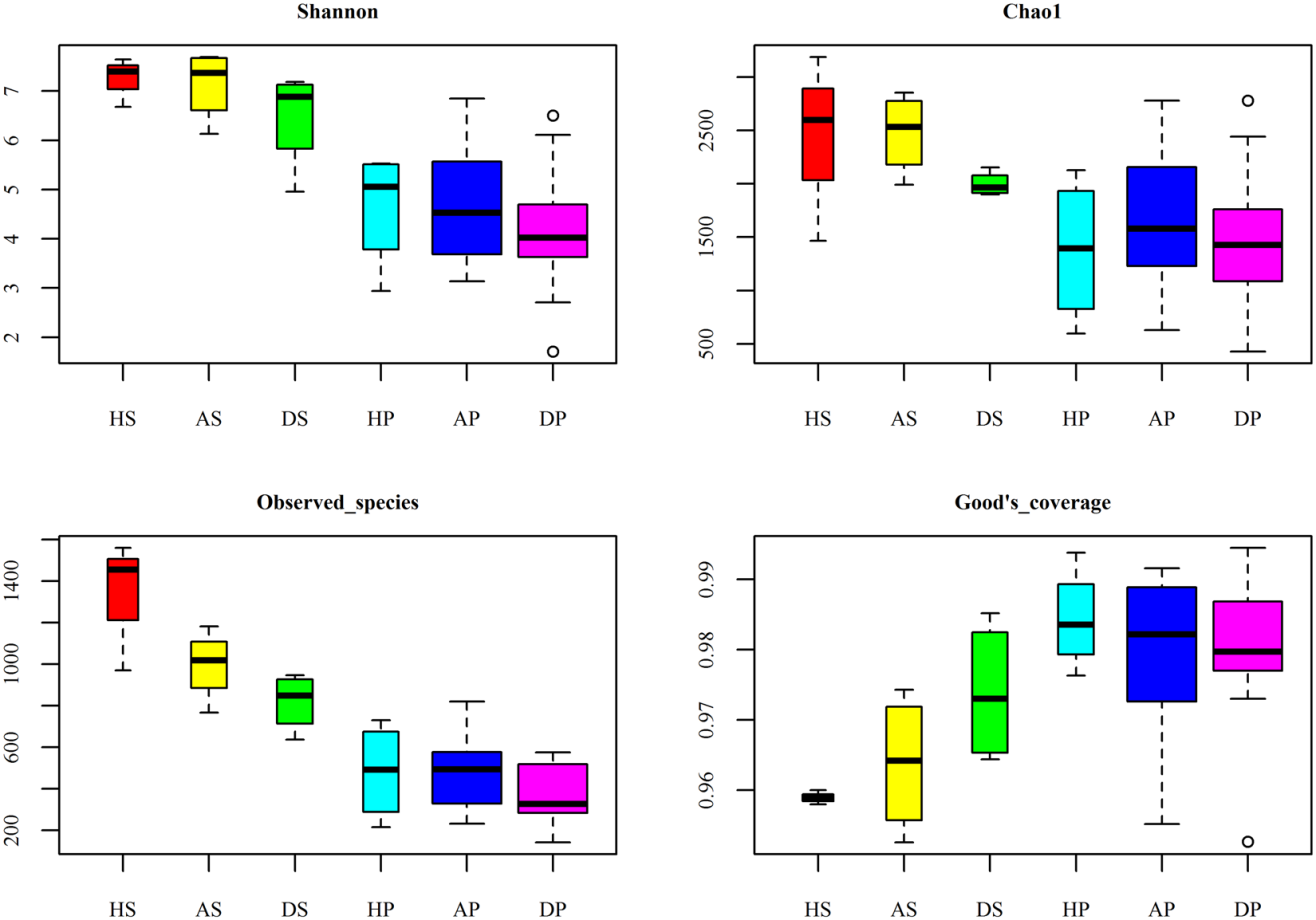
Boxplot of operational taxonomic units (OTUs) of Shannon, chao1, observed species, and Good’s coverage indices. The OTU similarity threshold of 97% was considered, and plateaus occurred at about 5,000 reads per sample.

Thirty-four newborn piglets under different healthy conditions (healthy piglet (HP, N = 4), asymptomatic piglet (AP, N = 15), and diarrheal piglet (DP, N = 15)) along with their respective mothers (health sow (HS, N = 3), asymptomatic sow (AS, N = 4), and diarrheal sow (DS, N = 4) were sampled. The 45 samples produced a total of 2,152,604 raw pair-end 300-bp reads from the MiSeq runs. After quality filtering and demultiplexing, 1,674,142 clean reads were obtained with an average of 31,363 ± 6,030 (s.d.), 35,900 ± 18,683 (s.d.), 38,778 ± 19,481 (s.d.), 42,134 ± 12,334 (s.d.), 40,586 ± 15,870 (s.d.), and 33,601 ± 8,339 (s.d.) clean reads in groups of HS, AS, DS, HP, AP, and DP, respectively (Table 1). After chimera filtering, OTUs were assigned by using pick_open_reference_otus.py script against Greengenes database 2013–08 release with 97% similarity. Of the 1,674,142 clean reads, 1,468,146 reads (87.70%) were clustered into 6,983 OTUs, and 205,996 (12.30%) reads were unassigned (Table S2). The low abundance OTUs under 0.005% cutoff were removed according to the criteria set by a previous report^32^. To reduce the bias generated by sequencing depth, the reads of each sample were normalized to 15,400 by QIIME script single_rarefaction.py (the minimum read number of the samples assigned to OTUs was 15,400 in this study) (Table 1). Alpha diversity indices, including Shannon, Chao1, Good’s coverage, Observed species, and Simpson, were introduced to describe the sequencing data in clinical samples (Fig. 1 and Table S3). Rarefaction curves of these indices plateaued a saturation phase after 5,000 reads per sample, suggesting adequate reads of all samples for the microbiota investigation (Fig. S1). The values of Good’s coverage of all these subjects were >95%, indicating sufficient sequencing depth for the investigation of gut microbiota in the six groups in this investigation.

### Relative analysis on GM structure and abundance between healthy sows and piglets

**Figure 2:**
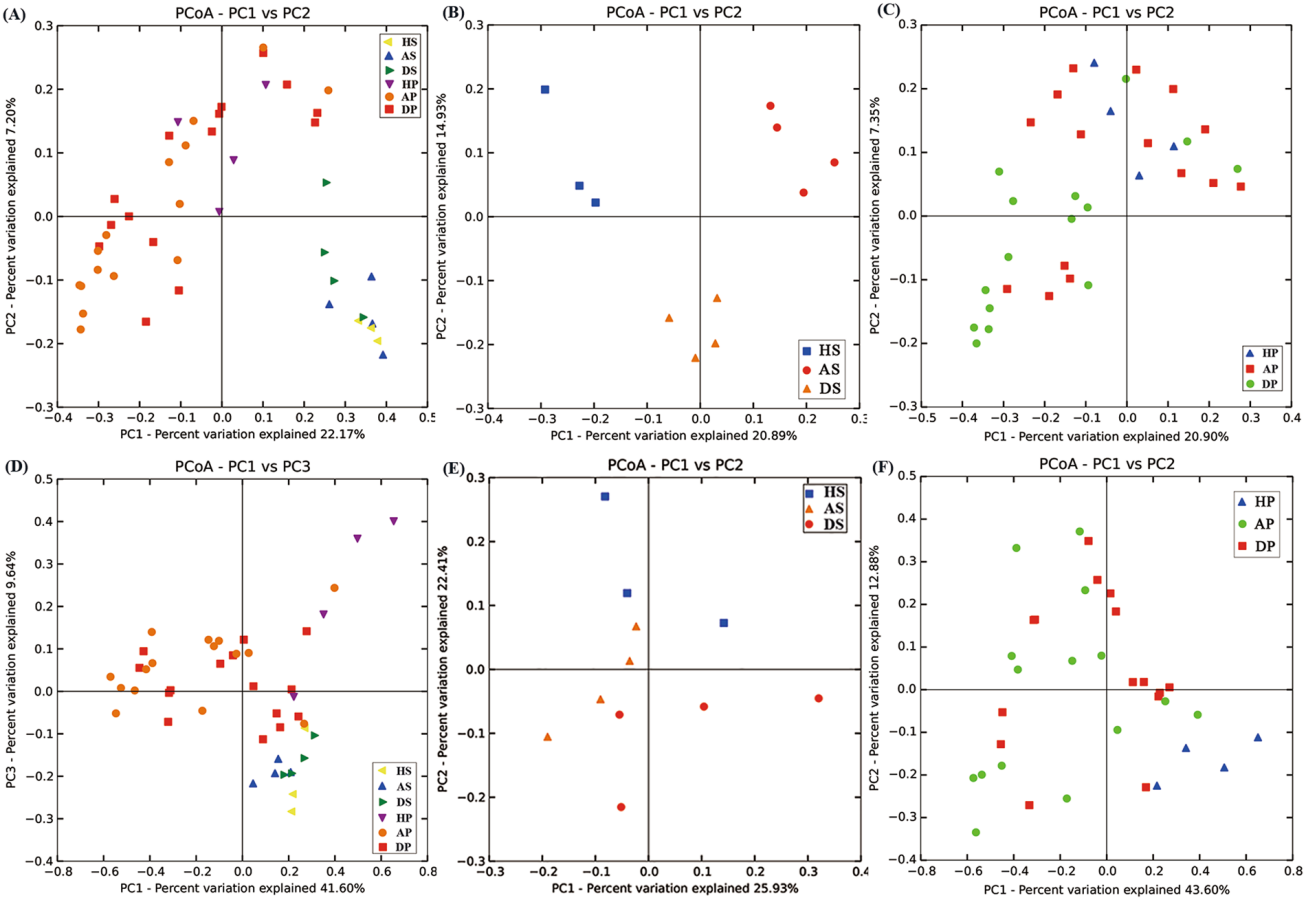
PCoA of the unweighted and weighted UniFrac distances for each group. Panel A, B and C showed the unweighted UniFrac distances of all the pigs sows and piglets, respectively. Panel D, E and F presented the weighted UniFrac distances of all the pigs, sows and piglets, respectively. The percent variation explained by each principal coordinate is indicated on the X and Y axes.

The GM structure of HS showed different alpha indices from that of HP: the HS GM have significant higher Shannon index (7.24 ± 0.50 s. d.), observed species (1328 ± 315 s. d.) and good’s coverage (0.96 ± 0.001 s. d.) than those of HP (4.65 ± 1.21 s. d., p = 0.019; 482 ± 235 s. d., p = 0.009; 0.98 ± 0.007 s. d., p = 0.002); while no marked difference was observed in Chao1 (Fig. 1 and Table S3). Principal coordinates analysis (PCoA) based on unweighted UniFrac distances revealed sharp segregations of GM structure between sows and piglets (Fig. 2A). Similar discrimination was observed under the PCoA by weighted UniFrac distances (Fig. 2D).

At the phylum level, the percentiles of dominant phyla of gut microbes in HS were 47.39% for *Bacteroidetes*, 34.57% for *Firmicutes*, 5.74% for *Proteobacteria*, 4.64% for *Spirochaetes*, 4.05% for *Tenericutes*, and 1.36% for *Verrucomicrobia*, respectively, while in HP, the abundances of these bacteria were 76.29%, 21.18%, 2.12%, 0.02%, 0.03%, and 0%, respectively (Table S4a). *Fibrobacteres*, *Planctomycetes*, *Cyanobacteria*, *Actinobacteria*, *Lentisphaerae*, *Thermi*, *Fusobacteria*, and *Synergistetes* were in low abundances in both HS and HP samples. The HS had significant higher abundances of *Proteobacteria*, *Spirochaetes*, *Tenericutes*, *Verrucomicrobia*, and *Fibrobacteres* than that in HP, while the HP showed a very high abundance of *Bacteroidetes*. At the genus level, an even sharper segregation of GM structure was observed between HS and HP. The HP had 15.29% unclassified OTU at genus level. In contrast, unclassified OTU in the HS was as high as 60.11%. *Bacteroides*, the most abundant genus in HP, accounted for about half of the abundance on genus level in HP, while there was only 1.39% in HS (Table S4b). A greater percentage of *Prevotella*, *Parabacteroides*, and *Lactobacillus* were observed in HP, while *Oscillospira*, *YRC22*, *CF231*, *Streptococcus*, *Ruminococcus*, and *Treponema* were higher in HS. These discrepancies on GM structure and abundance might explain the segregations on PCoA.

### Shifts in gut microbial diversity and abundance in healthy, asymptomatic and diarrheal sows

A total of 3,403 OTUs were clustered based on the 327,099 non-chimeric reads from sow samples (HS: 1,686; AS: 1,743; DS: 1,528). No significant changes were observed in Shannon, Chao1 and Simpson indices among the three groups (Table S3). The observed species was significantly lower in DS than that in HS (p < 0.05). The unweighted and weighted UniFrac distances revealed sharp segregations among these groups, indicating a core division in GM community and abundance among HS, AS and DS samples (Fig. 2B and E). Meanwhile, the UPGMA tree also showed the similar result (Fig. S2A).

*Bacteroidetes*, *Firmicutes*, *Proteobacteria*, *Spirochaetes*, and *Tenericutes* in HS, AS and DS were the predominant phyla, accounting for more than 96% of the overall abundance of bacteria (Table S5a), and each of the remaining phyla accounted for <3% in relative abundances. A significant increase in the ratio of *Firmicutes/Bacteroidetes* was observed in AS when compared to that in HS (p < 0.05). As compared, the ratio of *Firmicutes/Bacteroidetes* in DS was irregular and showed a slight higher than that in HS on average. A significant increase of *Actinobacteria* was found in AS, represented by *Coriobacteriaceae*. The abundance of *Proteobacteria* was decreased from HS to AS and DS, while the percentage of *Spirochaetes* was increased. Despite a small quantity of *Verrucomicrobia*, a trend of reduction was observed from HS to AS and DS.

**Figure 3:**
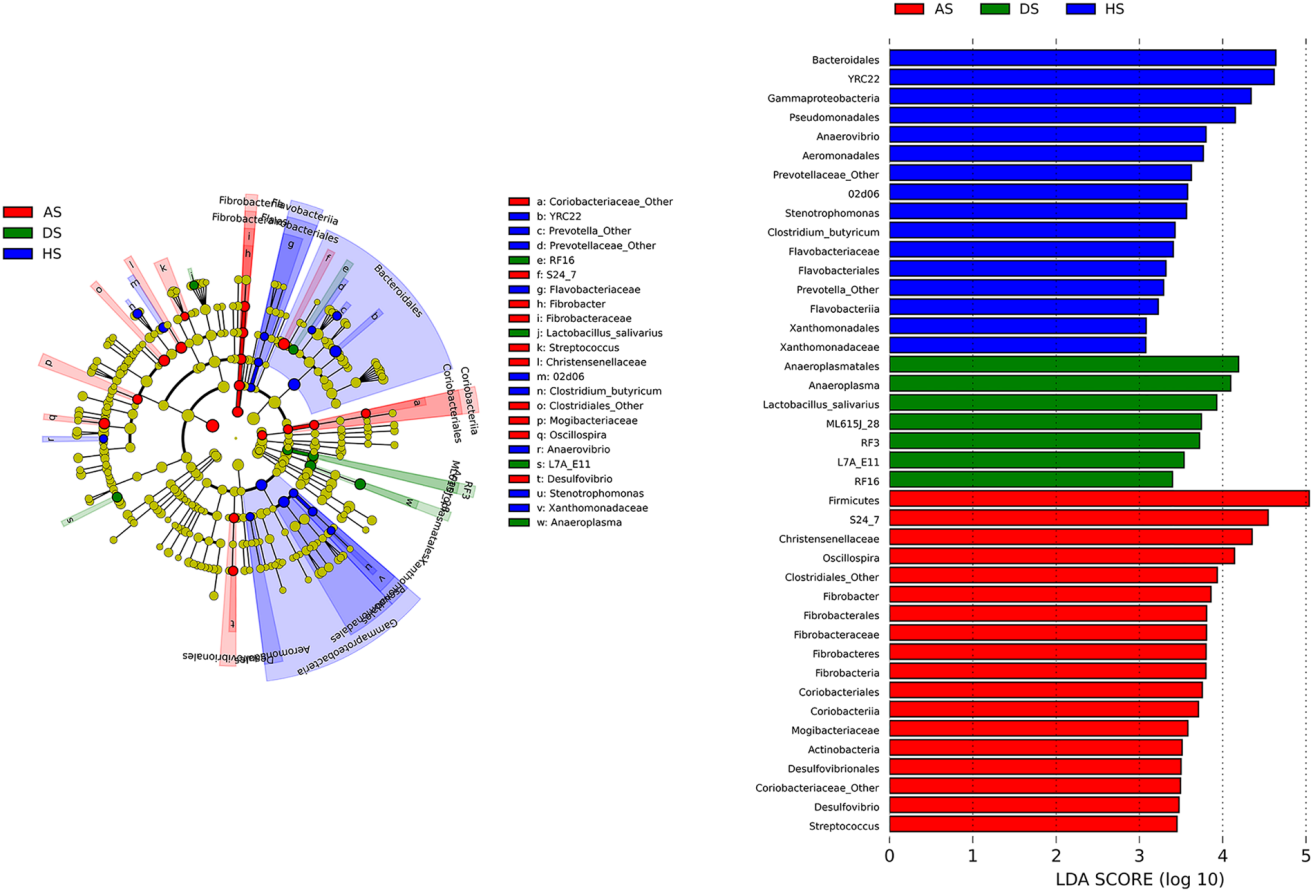
LEfSe identified taxons for HS, AS and DS. (Left) Taxonomic cladogram representation of statistically and biologically consistent differences among healthy, asymptomatic and diarrheal sows. Taxa enriched in HS, AS or DS are colored by blue, red and green, respectively. (Right) Histogram of the LDA scores computed for features differentially abundant among healthy, asymptomatic and diarrheal sows. Only taxa meeting an LDA significant threshold >2 and p < 0.05 are shown. The HS-, AS-, and DS-enriched taxa are shown with blue, red and green bars.

To identify specific biomarkers of GM in samples from HS, AS, and DS, the high-dimensional class comparison method of linear discriminant analysis (LDA) effect size (LEfSe) was employed to evaluate the statistical difference of GM abundance from the taxonomic phylum to species. A cladogram representing the structure of the host-microbiota axis showed significant shifts of GM among HS, AS and DS. A total of 41 phylotypes were discovered as high-dimensional biomarkers for separating GM among HS, AS and DS samples. Sixteen of these phylotypes relatively enriched in abundances in HS group. Of which, seven belonged to phylum *Bacteroidetes*, six were in *Proteobacteria*, and three in *Firmicutes*, respectively; eighteen phylotypes in phyla *Firmicutes*, *Bacteroidetes*, *Fibrobacteres*, and *Actinobacteria* were dominant in AS; and seven phylotypes in phyla *Tenericutes* and *Firmicutes* were leading phylotypes in DS (Fig. 3 and Table S6a). Interestingly, a butyrate-producing bacteria *Clostridium butyricum* was significantly higher in abundance in HS samples than that in AS and DS samples (p = 0.028). The family *Desulfovibrionaceae* and genus *Desulfovibrio*, which is positively associated with inflammation, were observed in higher percentage in AS than that in HS and DS groups.

The diarrheal sows had a significantly different GM structure compared to that in the healthy sows (Fig. S3A and Table S6b). Fifty-four phylotypes were found significantly different between HS and DS, of which 45 phylotypes were significantly higher in HS samples, and 9 were dominant in DS samples. The differential species in DS samples were mostly in the order *Clostridiales* in *Firmicutes* and family *Peptostreptococcaceae* in *Tenericutes*. When an overall difference of GM between HS and AS groups was compared, 31 phylotypes were found higher and 7 were lower in the HS group than the AS group (Fig. S3B and Table S6c). Most of the preponderant bacteria in HS fell into phyla *Bacteroidetes* and *Proteobacteria*, while the dominant GM in AS samples fell into phyla *Firmicutes* and *Actinobacteria*. The HS gut had a significantly different microbial structure with that in the DS samples (Fig. S3C and Table S6d). A total of 36 phylotypes were found to be different between AS and DS groups, with 28 higher in AS samples and 7 higher in DS samples. The AS had more *Verrucomicrobia* in GM, while the DS had more *Tenericutes*.

### Structure of gut microbiota in healthy, asymptomatic and diarrheal piglets

A total of 1,079 OTUs in all of the piglet samples (HP: 621, AP: 697, and DP: 519) were generated from 1,259,623 clean reads. Unexpectedly, no significant differences in alpha diversity indices among the groups were observed (Fig. 1 and Table S3). The unweighted UniFrac distance was not able to separate the HP, AP and DP samples (Fig. 2C); while the weighted UniFrac could slightly separate the HP samples from AP samples as well as the DP samples, indicating a difference in GM abundance between HP and AP/DP samples (Fig. 2F). The UPGMA tree could discriminate HP from other two groups, while couldn’t separate AP and DP samples (Fig. S2B). These beta diversity discrepancies between HP and AP/DP revealed the HP had different GM structure and composition than that of AP/DP samples.

The five most abundant phyla, *Bacteroidetes*, *Firmicutes*, *Proteobacteria*, *Fusobacteri*a, and *Actinobacteria*, accounted for over 99% of all piglet subjects. The remaining phyla accounted for < 1% abundance of bacteria (Table S5b). In particular, the GM in HP samples were largely dominated by *Bacteroidetes* (78.80% ± 16.38%, s. d.), significantly higher than those in AP and DP samples (p = 0.000), while the AP and DP samples contained a large number of taxa belonging to phylum *Fusobacteria* (AP: 15.36% ± 25.59%, s. d.; DP: 14.23% ± 24.65%, s. d.). Additionally, the AP and DP samples were remarkably different from HS with respect to high abundances of phylum *Proteobacteria* (AP: 16.43% ± 27.34% s. d., and DP: 12.74% ± 15.87% s. d.). At the genus level, about half of the reads from HP samples were assigned to *Bacteroides* (49.35%), followed by *Prevotella* (12.28%), *Lactobacillus* (8.21%), and *Oscillospira* (1.29%). The AP and DP samples showed remarkable differences in microbiota diversity and relative abundance, and the highest abundance of bacteria in both AP and DP samples was *Fusobacterium* (AP: 18.08%, DP: 15.28%), followed by *Lactobacillus, Bacteroides, Prevotella, Sutterella, Veillonella, Parabacteroides, Oscillospira*, and *Clostridium* in AP samples, and *Lactobacillus, Bacteroides*, *Clostridium*, *Prevotella*, *Veillonella*, *Ruminococcus*, *Campylobacter*, *Streptococcus*, *Collinsella*, *Oscillospira*, *Treponema*, *Escherichia*, *Peptostreptococcus*, and *Enterococcus* in DP samples.

**Figure 4:**
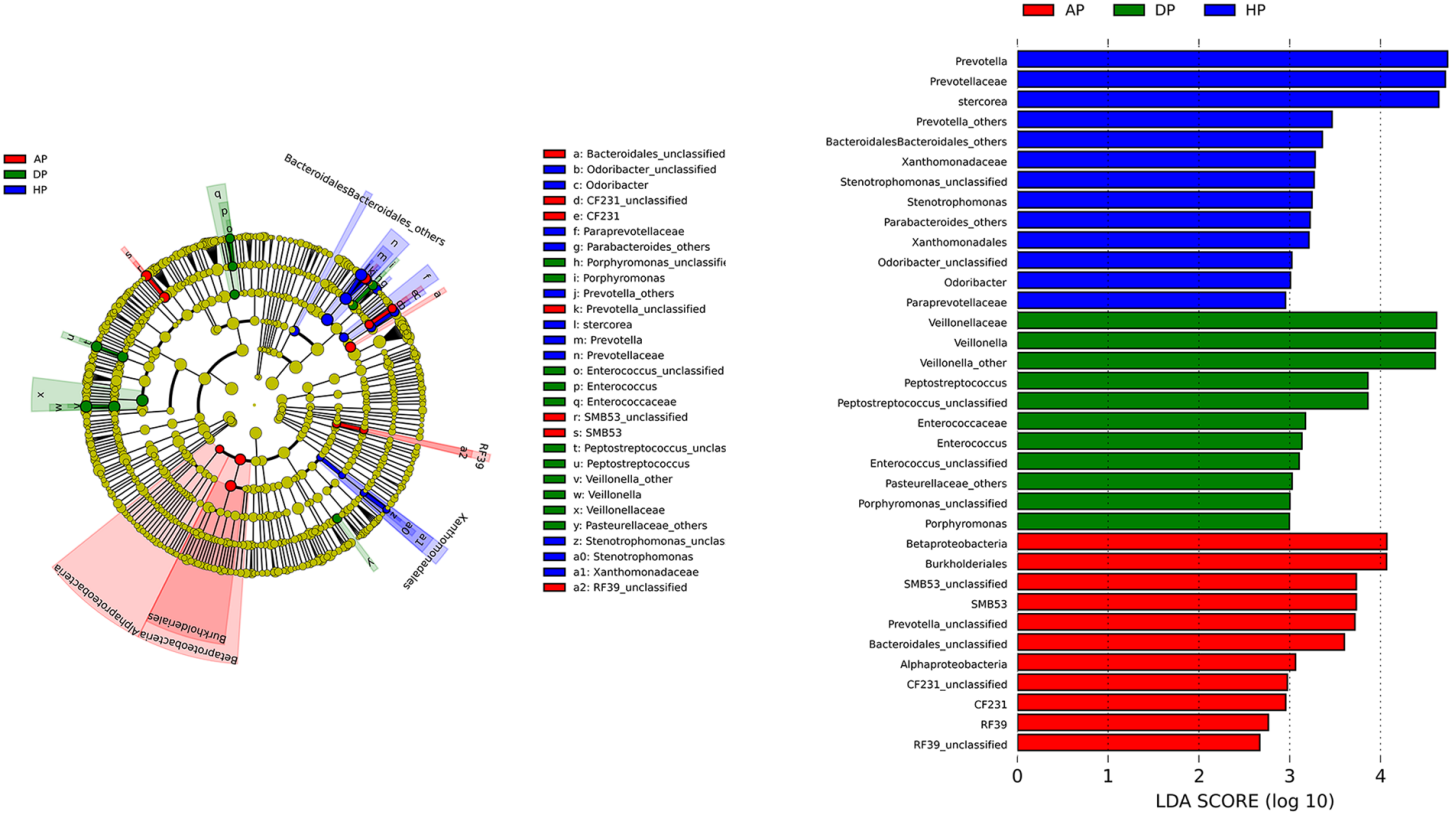
Differences in phylotypes of gut microbiota responding to piglets under different healthy status identified using LEfSe. (Left) Taxonomic cladogram representation of statistically and biologically consistent differences. Taxa enriched in HP, AP or DP are colored by blue, red and green, respectively. (Right) Histogram of the LDA scores. The HP-, AP-, and DP-enriched taxa are shown with blue, red and green bars.

LEfSe algorithm on microbial abundances of GM in piglets showed significant differences between the normal, asymptomatic and diarrheal piglets. Among the three groups, 35 phylotypes from phylum to species were discovered as high-dimensional biomarkers (Fig. 4 and Table S6e). The HPs had substantial higher abundances of GM in phyla of *Bacteroidetes* and *Proteobacteria*, due to an increase of *Prevotella*, *Odoribacter*, and *Parabacteroides* in phylum *Bacteroidetes*, and *Stenotrophomonas*, in phylum *Proteobacteria*, respectively; the DPs had distinct GM compositions mostly belonging to phyla *Firmicutes*, represented by *Enterococcus*, *Veillonella*, and *Peptostreptococcus* at genus level; while the APs had GM which mostly fell into phyla *Bacteroidetes*, *Tenericutes* and *Proteobacteria*, mainly contributed by *CF231*, unclassified *Prevotella*_, and *SMB53*.

A total of 24 phylotypes were significantly different between HP and AP samples. Of which two were overrepresented in HPs and the remaining 22 phylotypes were reduced in APs. Interestingly, the butyrate-producing bacterium, *Clostridium butyricum*, was overrepresented in the GM in HP samples. In contrast, the pathogenic *Clostridium perfringens* was overrepresented in the GM in AP group (Fig. S4A and Table S6f). Fifty one phylotypes showed significant differences between the GM of HP and DP samples (Fig. S2B and Table S6g). The biomarkers for separating GM in HP and DP groups at the phylum level were *Bacteroidetes* and *Firmicutes*, as the HP samples had higher abundance in *Bacteroidetes* in GM while the DP samples had more *Firmicutes* (Fig. S4B and Table S6g). The bacteria in genera *Bacteroides* and *Prevotella* made a significant contribution to the predominant phylum of *Bacteroidetes* in HP samples, while *Clostridiales* and *Enterococceae* highly contributed to the increase of *Firmicutes* in DP samples. Interestingly, *Clostridium butyricum*, a species in genus *Clostridium*, was the predominant phylotype in GM of HP group, while potential pathogenic *Clostridium perfringens*, another species in the same genus, showed higher abundance in the GM of DP group. Compared to the DP samples, the AP samples had 18 distinct phylotypes with either higher or lower abundances (Fig. S4C and Table S6h).

## Discussion

In this study, we determined and compared the fecal microbiome in healthy, asymptomatic, diarrheal sows and their piglets associated with PEDV infection. We systematically investigated the microflora profiles in sows associated with PEDV infection, but also evaluated the bacterial alternations from their healthy, asymptomatic and diarrheal offspring associated with PEDV infection. To our knowledge, this is the first report of using next-generation sequencing technology to compare the gut microbiota in asymptomatic and diarrheal sows naturally infected with PEDV, and their newborn piglets as well. During the outbreaks of severe diarrhea in pigs in 2010, we observed different clinical presentations between sows and suckling piglets, especially the neonates within 10 days old, in the field. Sows manifested a mild diarrhea which last for about one to two days, accompanied with mild anorexia and listlessness. However, neonates aged within 10-day had a severe diarrhea along with dehydration, vomiting and high morbidity and mortality as previously reported^1,9^. Diarrhea in neonates infected by PEDV could last for over 7 days and the treatment of antibiotics is usually inactive, and most of them (50–80%) died from dehydration within a week after infection. This urges us to explore underlying reasons responsible for the huge discrepancies in clinical symptoms and outcomes between PEDV-infected sows and piglets.

The gastrointestinal tract ecosystem is complex, and plays critical role in both health and disease^11,33^. The bacteria colonized in this niche is dynamic and subject to changes based on time, age, breed and many other factors^34^. It has been reported that *Firmicutes* and *Bacteroidetes* are the dominant phyla in mammal’s gut regardless of fluctuation and influence from multiple factors such as diseases, age, breed, diet, and gender^35^. In this study, we found that phyla *Bacteroidetes* and *Firmicutes* were the predominant phyla in the GM from HS, AS, DS, and HP, which was consistent with the aforementioned study. Changes in microbial memberships and proportions were observed due to the infection of PEDV, although the infected sows had just showed one- or two-day diarrheal disease. Furthermore, interesting membership shifts were detected in sows infected with PEDV. *Clostridium butyricum* was decreased in proportion of AS and DS (p = 0.028). This bacterium could produce short chain fatty acids (SCFAs), mainly butyrate, and contributes to complex carbohydrate breakdown in the gut, which is greatly related to colonic health^36,37^. While the family *Desulfovibrionaceae* and genus *Desulfovibrio* had been previously found to be positively associated with inflammation, and also showed inhibition of butyrate oxidation, resulting in disruption of intestinal barrier function^38^. The increased abundance of family *Desulfovibrionaceae* and genus *Desulfovibrio* in AS and DS might lead to inflammation in musoca and facilitated the infection of PEDV. Liu *et al*. (2015) reported a diminishment of *Verrucomicrobia* in PEDV infected piglets compared to the healthy controls^28^. Despite small proportion of *Verrucomicrobia* in GM of sows, we also saw a similar trend of decrease of this phylum from HS to AS and DS. Similar to Liu’s study, we found the reduction of genus *akkermansia* accounts mostly for the phylum *Verrucomicrobia*. It was found that *Akkermansia* was associated with immune regulation by inducing Foxp3 regulatory T cells, which could attenuate tissue inflammation^39^.

The bacteria colonization in piglet intestine is believed to begin immediately after birth, and is mainly influenced by their mothers as previously reported^40^. The mother’s feces, skin, and the surrounding environment contain a large amount of bacteria, which are likely to be the source of intestinal microbiota of piglets^41^. The factors that have been recognized to be associated with enteritis and diarrhea in suckling piglets included viruses (*e. g*. rotavirus, coronavirus), bacteria (*Escherichia coli*, *Clostridium perfringens*, and *Clostridium difficile*) and parasites (*Cryptosporidium spp*., *Giardia spp*., *Cystoisospora suis* and *Strongyloides ransomi*). In this study, we found that the microbiota in the gut of neonates was greatly affected with PEDV infection. For instance, *Bacteroidetes* abundance was significant dropped from HP group (78.80% ± 17.33% s. d.) to both AP (23.05% ± 20.15% s. d.) and DP (21.09% ± 23.11% s. d.) groups. PEDV infection has also caused the increase of abundances in *Firmicutes*, *Proteobacteria*, and *Fusobacteria*, respectively. The significant increase in abundance of *Enterobacteriaceae* was the major reason for the increase of *Proteobacteria* in AP and DP groups. In addition, it was reported that *Fusobacteria* was greatly increased in the GM of PEDV-infected pigs^28,29^. A similar finding, a sharp rise of *Fusobacteria* abundance in GM of AP and DP, was also observed in GM of piglets in this study. However, the increased abundance of genus *Fusobacterium* was the key contribution for the rise of phylum *Fusobacteria*, which was in accordance with Koh’s report (2015)^28^, but inconsistent with Liu’s findings (2015)^27^. *Fusobacteria*, as typically dominant obligate anaerobic bacteria clustered in mouth, have been identified in a wide range of clinical anaerobic infections including oral, dental, gut and brain, and correlated positively with catarrhal appendicitis of mucosal surface^42–44^. Recently, it was reported that *Fusobacterium* plays a vital role in facilitating colorectal cancer^45^, and *Fusobacterium nucleatum* could definitely cause intestinal inflammation^46^. Perhaps the proportion increase of *Fusobacterium* in AP and DP could cause bowel inflammation. We observed a higher abundance of *Bacteroidetes* in GM of piglets than that in sows, which confirmed a previous study that the *Bacteroidetes* decreased as the swine gain weight and aged^47^. Our observations of decreased *Bacteroidetes* and increased *Firmicutes* amount and the ratio of *Firmicutes/Bacteroidetes* in asymptomatic and diarrheal samples from both sows and piglets are in agreement with a recently published study on pigs^47^. The bacteria in phylum *Bacteroidetes* are dominant bacteria of GM in healthy piglets, which includes those with high proportions in the predominant genera *Bacteroides*, *Prevotella*, and *Parabacteroides*. It is presumed that the shifts in GM composition and abundance correspond to the changes of diet and essential metabolic functions^48^. Another healthy associated bacterium, *Clostridium butyricum*, was significantly higher in abundance in HP than that in AP and DP. Studies revealed that *Prevotella*, *Bacteroides*, and *Coprococcus* are short-chain fatty acid producing bacteria and *Clostridium butyricum* are butyrate-producing bacteria. Therefore, the decreased proportion of these bacteria may lead to the de-stabilized of gut microbiome and may facilitate the infection of PEDV. In addition to the decrease of probiotic bacteria in asymptomatic and diarrheal pigs, we observed the increase of pathogenic bacteria in these infected pigs. Recently, *Clostridium perfringens* and *Clostridium difficile* were found to be ones of the most common bacterial species associated with diarrhea in pigs worldwide^49^. In this study, the relative abundances of *Clostridium perfringens* in both diarrheal sows and piglets were 10 to 15 times higher than those of healthy piglets. The *Clostridium perfringens* type C is rarely found in intestine of healthy piglets, but as a primary pathogen, it can also colonize on gut following other pathogens, such as transmissible gastroenteritis virus (TGEV), rotavirus, and PEDV^49^. *Clostridium perfringens* might be also as a primary pathogen to advance the infection of PEDV. Following the infection of PEDV, it may aggravate the PED as a secondly colony. Unlike sows, a decrease trend in proportion of *Verrucomicrobia* was found in the HP samples when compared to that in AP and DP samples. To date, there are no reports of a relationship between hosts and *Verrucomicrobia*. Dubourg *et al*.^50^ reported an increase level in the colonization of human gut by *Verrucomicrobia* due to dysbiosis, i.e., microbial imbalance of gut microbiota after broad-spectrum antibiotic treatment. Therefore, the *Verrucomicrobia* detected in the swine gut might be due to the use of antibiotics, since antibiotics are usually used in pig farms for preventive or therapeutic purposes.

Unexpectedly, we observed a high relative abundance of *Bacteroides fragilis* in all three piglet groups. A similar finding was previously reported in young children with diarrhea^16^. *Enterococci* were presented in both asymptomatic and diarrheal samples, including AS and AP. However, none of the sequences from HS and HP samples were assigned to this genus. *Enterococcus* is a large genus of Gram-positive lactic acid bacteria of the phylum *Firmicutes*, commonly resident in the intestines of humans and animals, including the pig^51^. Important clinical infections caused by *Enterococcus* include urinary tract infections, bacteremia, bacterial endocarditis, diverticulitis, and meningitis^52^. It has been reported that there is an association of *Enterococci* to neonatal porcine diarrhea, and also association with resistance to several antibiotics^53,54^, which could explain why the inefficiency of antibiotic treatment of these diarrheal piglets.

**Table 1:** Reads summary of clinical samples.

Group	Total Clean Reads	Average clean reads	Reads assigned to OTU	OTUs
HS	94,089	31,363 ± 6,030	24,553 ± 5,200	1862
AS	143,599	35,900 ± 18,683	29,640 ± 14,689	1743
DS	155,111	38,778 ± 19,481	33,721 ± 17,861	1528
HP	168,535	42,134 ± 12,334	37,993 ± 10,585	1258
AP	608,791	40,586 ± 15,870	35,851 ± 14,761	697
DP	504,017	33,601 ± 8,339	30,087 ± 7,792	519

Note: healthy pigs: showing no clinical symptoms of diarrhea and free of common diarrheal pathogens tested by PCR, qPCR and sequencing; asymptomatic pigs: showing no clinical symptoms of diarrhea but PEDV positive (mono-infection alone) tested by PCR/RT-PCR and qPCR/qRT-PCR; and diarrhea pigs: showing clinical symptoms of diarrhea and PEDV positive (mono-infection alone) tested by PCR/RT-PCR and qPCR/qRT-PCR.

## Conclusion

In conclusion, our study demonstrates that dysbiosis in gut microbiota occurs in asymptomatic and diarrheal sows and their piglets. This dysbiosis caused by PEDV infection resulted in unbalance and translocation of gut microbiota from phylum to genus, or even species levels. The abundances of inflammatory or diarrhea associated pathogenic bacteria were significantly increased while the beneficial bacteria, e.g., butyrate-producing bacterium, were reduced in asymptomatic and diarrheal sows. PEDV infection also switched the dominant bacteria in gut of piglets. *Fusobacteria* and *Proteobacteria* were remarkably increased in AP and DP, while the abundances of short-chain fatty acid producing bacteria and the butyrate-producing bacterium were remarkably decreased in DP and AP. Our findings from this study demonstrated that the gut microbiota were altered due to PEDV infection, which caused the severe perturbations of GM in both sows and piglets through diminishing probiotic bacteria and enriching pathogenic bacteria.

## Materials and Methods

### Samples

The ethics committee of Jiangxi Agricultural University approved the animal protocol for this study (protocol number P-2013–03). All the procedures involving animals in this study were carried out in accordance with *The care and use guidelines of experimental animals* established by the Ministry of Agriculture of China.

Mock community of bacteria. The mock community (MC) of bacteria were prepared by pooling equimolar genomic DNA of each species of 16 known bacteria, following the instructions in the previous study (Detailed information is in Appendix 1. Experimental Procedures).

Clinical samples. Clinical samples were collected from December 2013 to March 2014, from an independent commercial farm with about 600 sows in Jiangxi, China, which suffered with severe diarrhea (for details, please refer to in the supporting information, SI). Thirty-four piglets under different healthy conditions (healthy piglets (HP, N = 4); asymptomatic piglets (AP, N = 15), and diarrheal piglets (DP, N = 15), were sampled, along with their respective mothers (health sow (HS, N = 3), asymptomatic sow (AS, N = 4), and diarrheal sow (DS, N = 4). Fresh feces were immediately collected, transported to our laboratory on ice and then stored at −80°C until use. The details on study design are described in Supporting Information, SI.

### DNA extraction, 16 S rRNA gene amplification, and high-throughput sequencing

Total DNA of each sample was extracted from aliquots of frozen feces (200 mg, 45 samples) using the QIAamp Stool DNA Extraction Kit (Cat. no. 51504, QIAGEN, Germany) according to the manufacturer’s instructions, and quantified using NanoDrop 2000 UV spectrophotometer (NanoDrop Technologies, USA) and gel electrophoresis. Amplification of the V1-V3 hypervariable regions of 16 S rRNA gene was performed by using ‘universal’ primers 27 F/534 R (27 F: 5′-AGAGTTTGATCCTGGCTCAG-3′, 534 R: 5′-ATTACCGCGGCTGCTG G-3′)^55^ flanked with adapters, and a sample-unique 12-mer sequences of barcode between adapter and primer sequences of 534 R. For each sample, 500 ng purified DNA was used for library construction by using Illumina TruSeq^®^ DNA PCR-Free Sample Preparation Kit (Illumina, San Diego, CA), and then were sequenced on Illumina MiSeq platform running by 300 bp paired-end methodology.

### Data processing, bioinformatics analysis and statistical analysis

The raw reads were put for quality control after base calling, and then were subjected to pipeline QIIME v1.9.0 (http://qiime.org/tutorials/illumina_overview_tutorial.html) for OTU picking and taxonomy^56^. Alpha and beta diversities were also analyzed by UPGMA and PCoA (for details, see Appendix 1. Experimental Procedures). An algorithm for discriminating high-dimensional biomarker of genomic features, LEfSe (http://huttenhower.sph.harvard.edu/galaxy) was used to discover and explain the differences of microbiome from phylum to species levels between PEDV infected and non-infected groups as described previously^57^. The R packages Vegan, multcomp and MASS were used to perform statistical analysis. ANOVA was used to test the difference of alpha diversity indices and OTU/bacteria abundances between samples by using R package multcomp^58^.

## Electronic supplementary material

Combined Supplemental information
