# Supplementary material for: Altered Gut Microbiota Profiles in Sows and Neonatal Piglets Associated with Porcine Epidemic Diarrhea Virus Infection
Source: Sci Rep. 2017 Dec 12;7:17439. doi: 10.1038/s41598-017-17830-z (PMC5727058; doi:10.1038/s41598-017-17830-z)
Supplement: Supplementary file 1 — Combined Supplemental information [file 41598_2017_17830_MOESM1_ESM.pdf]

Altered Gut Microbiota Profiles in Sows and Neonatal Piglets Associated with Porcine Epidemic Diarrhea Virus Infection

Running title: Altered Gut Microbiota Profiles to PEDV Infection

Deping Song[1, 2], Qi Peng[1, 2], Yanjun Chen[1, 2], Xinrong Zhou[1, 2], Fanfan Zhang[1, 2], Anqi Li[1, 2], Dongyan Huang[1, 2], Qiong Wu[1, 2], Yu Ye[1, 2], Houjun He[1, 2], Leyi Wang[3]*, Yuxin Tang[1, 2]*

[1] Key Laboratory for Animal Health of Jiangxi Province, Nanchang, Jiangxi, 330045, China

[2] Department of Preventive Veterinary Medicine, College of Animal Science and Technology, Jiangxi Agricultural University, Nanchang, Jiangxi, 330045, China

[3]Department of Veterinary Clinical Medicine and the Veterinary Diagnostic Laboratory, College of Veterinary Medicine, University of Illinois, Urbana, IL, 61802, United States of America.

* Corresponding authors

E-mail: tang53ster@gmail.com (YT), leyiwang@illinois.edu (LW)

Appendix 1. Experimental Procedures

Detailed information of clinical samples.

Sows were puerperal Yorkshire-Landrace crossbred and then were cross-bred with Duroc boar, delivering Yorkshire-Landrace-Duroc crossbred commercial pigs. From October 2012 to March 2014, the farm has experienced at least thrice outbreaks of severe diarrhea in suckling piglets within 10 days, as well as temporary diarrhea in sows and finishing pigs. Suckling piglets suffered diarrhea characterized with profuse watery diarrhea, vomiting, dehydration and mortality over 80%. About 300 diarrheal samples from the farm were examined by an established RT-PCR method in our lab, and 70% of these samples were identified as PEDV positive infection, resembling the disease caused by variant PEDV (data not shown). Based on the healthy status and age, samples were classified into six groups: (1) Sows without diarrhea during pregnancy and lactation and without detectable PEDV in feces were selected as healthy sows (HS), (2) sows without diarrhea during pregnancy and lactation, but the labors exhibited diarrhea less than 7 days were selected as asymptomatic sows (AP), (3) sows with diarrhea within a month until sampling were set as diarrheal sows (DS), (4) piglets aged in five days born from healthy sows and without diarrhea and detectable PEDV in feces were set as healthy piglets (HP), (5) piglets under the age of 10-day born from asymptomatic and diarrheal sows and without observation of diarrhea were set as asymptomatic piglets (AP), and (6) diarrheal piglets within 10-day old born from AS and DS were set as diarrheal piglets (DP).

Mock bacterial community (MC).

The bacteria for the mock community (MC) contained a variety of microorganisms isolated and identified by our lab. Most of the bacteria were isolated from pigs, including probiotics and pathogens. All of the MC bacteria have been identified by chemistry methods and Sanger sequencing on 16S rRNA (small subunit of the ribosome in bacteria), amplified by the universal

primer 8F/1492R (8F: 5'-AGAGTTTGATCCTGGCTCAG-3', 1492R: 5'-GGTTACCTTGTTACGACTT-3'). The bacteria included were *Bacillus natto, Bacillus subtilis, Bacillus licheniformis, Bacillus cereus, Clostridium perfringens, Lactococcus lactis, Lactobacillus rhamnosus GG, Staphylococcus aureus, Erysipelothrix rhusiopathiae, Haemopilus parasuis, Staphylococcus .sp, Proteus mirabilis, Enteropathogenic E.coli, Pseudomonas aeruginosa, Microbacterium lacticum, Trueperella pyogenes*. Genomic DNA of each bacterial strain was extracted and then quantified using NanoDrop 2000 UV spectrophotometer (NanoDrop Technologies, USA). DNAs of each bacterium were equimolar pooled to create MC. Each of the DNAs from MC was quantified by a SYBR Green quantitative RT-PCR (qRT-PCR) based on amplification of the 16S rRNA gene copy number. The universal primers 357F/519R (357F: 5'-CTCCTACGGGAGGCAGCAG-3', 519R: 5'-GWATTACCGCGGCKGCTG-3') were used, and the qRT-PCR was performed by the method described previously[1].

Sequencing consistency of MC.

To evaluate the primers for MiSeq sequencing and analytic workflows, a 'mock' community (MC) was constructed by 15 bacterial species belong to three phyla: *Firmicutes*, *Proteobacteria*, and *Actinobacteria* (Table S1a). The equimolar pooled MC DNA was amplified and sequenced by a MiSeq PE300 run together with that of clinical samples. Sequences were obtained by base-calling, filtered, and resulted in 52,516 clean reads. Reads were processed using package QIIME v 1.9.0, and 47,512 of the clean reads were generated into OTUs, and then OTUs were filtered based on the cutoff of <0.005% in abundance. Firstly, we examined the observed taxonomy consistency of reads from MiSeq run to the pooled MC. At phylum level, 99.27% of

the non-chimeric reads were assigned, and all belonged to *Firmicutes* (N=39,423), *Proteobacteria* (N=5,981), and *Actinobacteria* (N=1,760), only 330 reads were assigned to other three phyla (*TM7*, N=44; *Tenericutes*, N=163; *Spirochaetes*, N=23) and 118 reads were not assigned to any bacteria; at the genus level, 93.50% (N=44,422) of the assigned reads (N=47,512) were targeting 31 genera, with 6.50% of the OTUs were unclassified and/or others. Of the 31 genus generated, the 13 genus for the pooled DNAs of MC occupied 98.78% of the assigned reads (N=43,880), and the plus 542 assigned reads were targeting other 18 genera (Table S1b).

Secondly, we explored the consistence of relative abundance of each community member between the results of MiSeq sequencing and qPCR. We sought to establish the quantitative PCR (qPCR) to quantify the accurate community compositions of the MC, and to observe the consistency between qPCR and next generation sequencing (NGS) methods. There were differences in the relative abundance of the MC bacteria between the two methods: *Clostridium perfringens, Lactococcus lactis, Erysipelothrix rhusiopathiae, Haemopilus parasuis, Proteus mirabilis, Enteropathogenic E.coli, Pseudomonas aeruginosa*, and *Trueperella pyogenes* were overrepresented in NGS sequences (varied from 1.01 ~ 1.46 fold), while *Bacillus, Lactobacillus rhamnosus GG, Staphylococcus aureus, Staphylococcus .sp*, and *Microbacterium lacticum* were underrepresented (varied from 0.23 ~ 0.81 fold) (Table S1b). Taken overall MC into consideration, the MiSeq results were mostly consistent with the results from qPCR, the ratio of NGS abundance and qPCR was 0.99. In general, the primer (27F/534R) was proved accuratelyto amplify the 16S rDNA to represent the microflora and the methodology used in this study was appropriate for unveiling the microbial communities in

clinical samples.

Data processing and bioinformatics analysis.

The average quality score of the raw data was analyzed and evaluated by FastQC version 0.11.2 released (http://www. bioinformatics. babraham.ac.uk/)[2]**.** Adapters and nucleotides with quality <Q20 were cut by Cutadapt software[3]. Reads without barcode and/or primer sequence was removed. Furthermore, reads with three consecutive low quality base calls, with <75% high quality base calls and length, and reads with one or more N base were removed. The forward and reverse MiSeq pair-end reads were merged to assemble the V1~V3 contigs by FLASH program[4], samples were split according to the barcode sequences used in the primers of each sample, and primer and barcode sequences in 5'/3' end of each read was truncated. Reads were then subjected to package Quantitative Insights Into Microbial Ecology (QIIME) v1.9.0 for the following analyses: Chimeras were checked and filtered to create clean reads by using Usearch algorithm (v 6.1) with default settings[5, 6]. Operational taxonomic units (OTUs) were generated with the pick_open_reference_otus.py script against Greengenes database 2013-08 release with a threshold of 97% similarity by using PyNAST[7, 8]. OTUs were picked by PyNAST method using the algorithm of selecting the longest sequence among identical reads, and abundance of each OTU in the initial sequences was recaptured by counting the number or proportion of the identical reads. The most abundant member of each OTU was selected as the representative sequence of that OTU. OTU representative sequences were assigned to Greengenes database on taxonomic levels from phylum to genus, and even to species level, and then annotated by Ribosomal Project naive Bayesian classifier (v2.2, rdp.cme.msu.edu, release 10.4) at a 80% similarity threshold[9]. Taxonomic levels from phylum to species of each sample was generated

into a collection of tables by MEGAN4 software (http://ab.inf.uni-tuebingen.de/software/megan/), and rep_set.tree was constructed by species abundance and microbial community structure[10]. To explore OTUs/taxonomy that were ubiquitously present among samples, Venny (v2.0), an interactive online tool (http://bioinfogp.cnb.csic.es/tools/venny/index.html) was used.

To avoid the bias that may be introduced by sequencing depth for each sample, reads were normalized to 15,400 reads for each sample (we chose 15,400 as the highest sequence because the smallest non-chimeric reads in the subsamples is 15,400) with the single_rarefaction.py script within QIIME before being used for alpha and beta diversity analyses. Alpha diversity analysis was performed with QIIME scripts alpha_rarefaction.py and alpha_diversity.py. The dynamic of richness, diversity and evenness in microbiota of pigs was represented by indices of Shannon[11], Chao 1[12], observed species, and Simpson[13]. The R packages ggplot2 and igraph were used to draw figures on boxplot of alpha diversity indices and bacterial abundances.

Weighted and unweighted UniFrac distances were computed for beta diversity comparisons with QIIME script of beta_diversity_through_plots.py[14]. UniFrac distance metrics was used for PCoA and UPGMA to constructed phylogenetic trees visualized by FigTree v1.4.2[15-17]. Script make_2d_plots.py was used to generate 2-dimensional (2D) PCoA.

Data submission

The 16S rRNA sequences described in this study were deposited at Sequence Read Archive (SRA) database belongs to National Center for Biotechnology Infromation (NCBI) under the BioProject PRJNA401527 and NCBI BioSample SAMN07626583.

References:

1. Turner, S., Pryer, K.M., Miao, V.P.W., and Palmer, J.D. 1999. Investigating deep phylogenetic relationships among cyanobacteria and plastids by small subunit rRNA sequence analysis. Journal of Eukaryotic Microbiology 46: 327–338.

2. Schmieder, R. & Edwards, R. Quality control and preprocessing of metagenomic datasets. *Bioinformatics* **27**, 863-864, doi:10.1093/bioinformatics/btr026 (2011).

3. Martin, M. Cutadapt removes adapter sequences from high-throughput sequencing reads. *EMBnet. journal* **17**, pp. 10-12 (2011).

4. Magoc, T. & Salzberg, S. L. FLASH: fast length adjustment of short reads to improve genome assemblies. *Bioinformatics* **27**, 2957-2963 (2011).

5. Caporaso, J. G. *et al.* QIIME allows analysis of high-throughput community sequencing data. *Nature methods* **7**, 335-336, doi:10.1038/nmeth.f.303 (2010).

6. Kuczynski, J. *et al.* Using QIIME to analyze 16S rRNA gene sequences from microbial communities. *Current protocols in microbiology*, 1E. 5.1-1E. 5.20 (2012).

7. DeSantis, T. Z. *et al.* Greengenes, a chimera-checked 16S rRNA gene database and workbench compatible with ARB. *Applied and environmental microbiology* **72**, 5069-5072, doi:10.1128/AEM.03006-05 (2006).

8. Caporaso, J. G. *et al.* PyNAST: a flexible tool for aligning sequences to a template alignment. *Bioinformatics* **26**, 266-267 (2010).

9. Wang, Q., Garrity, G. M., Tiedje, J. M. & Cole, J. R. Naive Bayesian classifier for rapid assignment of rRNA sequences into the new bacterial taxonomy. *Applied and environmental microbiology* **73**, 5261-5267, doi:10.1128/AEM.00062-07 (2007).

10. Huson, D. H., Mitra, S., Ruscheweyh, H. J., Weber, N. & Schuster, S. C. Integrative analysis of environmental sequences using MEGAN4. *Genome research* **21**, 1552-1560, doi:10.1101/gr.120618.111 (2011).

11. Shannon, C. E. The mathematical theory of communication. 1963. *M.D. computing : computers in medical practice* **14**, 306-317 (1997).

12. Chao, A. Nonparametric estimation of the number of classes in a population. *Scandinavian Journal of statistics*, 265-270 (1984).

13. Hunter, P. R. & Gaston, M. A. Numerical index of the discriminatory ability of typing systems: an application of Simpson's index of diversity. *Journal of clinical microbiology* **26**,

2465-2466 (1988).
14. Lozupone, C. A., Hamady, M., Kelley, S. T. & Knight, R. Quantitative and qualitative β diversity measures lead to different insights into factors that structure microbial communities. *Applied and environmental microbiology* **73**, 1576-1585 (2007).
15. Hamady, M., Lozupone, C. & Knight, R. Fast UniFrac: facilitating high-throughput phylogenetic analyses of microbial communities including analysis of pyrosequencing and PhyloChip data. *The ISME journal* **4**, 17-27 (2010).
16. Lozupone, C., Lladser, M. E., Knights, D., Stombaugh, J. & Knight, R. UniFrac: an effective distance metric for microbial community comparison. *The ISME journal* **5**, 169 (2011).
17. Lozupone, C. & Knight, R. UniFrac: a new phylogenetic method for comparing microbial communities. *Applied and environmental microbiology* **71**, 8228-8235 (2005).

Supplementary Tables and Figures

Table S1. Background information and quantification of the MC used in this study. Each of the bacteria used was examined by chemistry test and sequenced the 16S rRNA by universal primers 8F and 1492R. a) Taxonomy of each bacterium from phylum to genus was annotated. 500 ng from each mock bacterium was pooled to create MC. b) Sequences were filtered to obtain clean reads and then were assigned to Greengenes database to generate OTUs. DNA concentration of each mock bacterium was quantitated by the qPCR assay by primer 357F/519R, and then the proportion of each mock bacterium was calculated based on the results from qPCR and MiSeq sequencing, respectively. The ratio of qPCR/MiSeq were then calculated to analyze the consistence of these two methods.

Table S2. Summary of study samples and fecal bacteria 16S rRNA amplicon sequence datasets.

Table S3. Statistics of alpha diversity indices in all clinical samples. The indices of Shannon, Chao1, Good's coverage, Observed species, and Simpson were introduced to describe alpha diversity of data in all clinic samples.

Table S4. Comparison of bacteria abundance between HS and HP at phylum and genus level

Table S5. The bacterial abundance at the phylum level were comparison by ANOVA among the sow (a) and piglet (b) groups.

Table S6. Relative differences in microbial taxa abundance in different groups. (a) HS, AS and DS; (b) HS and DS; (c) HS and DS; (d) AS and DS; (e) HP, AP and DP; (f) HP and AP; (g) HP and DP; (h) AP and DP.

Fig. S1. Rarefaction curves of the clinic sequencing data under the condition of 0.97 similarity. The observed species of each sample was in plateaued saturation phase over 5,000 reads.

Fig. S2. The unweighted pair group method with arithmetic mean (UPGMA) tree showing relationships among samples of sow (A) and piglet (B) according to bacterial profile.

Fig. S3. Histogram of the LDA scores computed for features differentially abundant among healthy, asymptomatic and diarrheal sows. (A) HS (colored in green) vs. DS (red); (B) HS (green) vs. AS (red); (C) AS (red) vs. DS (green).

Fig. S4. Histogram of the LDA scores computed for features differentially abundant among healthy, asymptomatic and diarrheal piglets. (A) HP (colored in green) vs. AP (red); (B) HP (green) vs. DP (red); (C) AP (red) vs. DP (green).

Table S1. Background information and quantification of the MC used in this study. Each of the bacteria used was identified by chemistry test and sequenced the 16S rRNA by universal primers 8F and 1492R. a) Taxonomy of each bacteria from phylum to genus was annotated. 500 ng from each mock bacteria was pooled to create MC. b) Sequences were filtered to obtain clean reads and then were assigned to Greengenes database to generate OTUs. DNA concentration of each mock bacteria was quantified by the qPCR assay by primer 357F/519R, and then the proportion of each mock bacteria was calculated based on the results from qPCR and MiSeq sequencing, respectively. And then the ratio of qPCR/MiSeq were calculated to analyze the consistence of these two methods.

a. Taxonomy of MC

Bacterial species	Taxon					Pooled genomic DNAs		
	Phylum	Class	Order	Family	Genus	DNA Conc. (ng/μl)	Volumes mixed (μl)	pooled DNA quanlity (ng)
Bacillus natto	*Firmicutes*	*Bacilli*	*Bacillales*	*Bacillaceae*	*Bacillus*	99.30	5.04	500.00
Bacillus subtilis	*Firmicutes*	*Bacilli*	*Bacillales*	*Bacillaceae*	*Bacillus*	154.80	3.23	500.00
Bacillus licheniformis	*Firmicutes*	*Bacilli*	*Bacillales*	*Bacillaceae*	*Bacillus*	162.60	3.08	500.00
Bacillus cereus	*Firmicutes*	*Bacilli*	*Bacillales*	*Bacillaceae*	*Bacillus*	126.20	3.96	500.00
Clostridium perfringens	*Firmicutes*	*Clostridia*	*Clostridiales*	*Clostridiaceae*	*Clostridium*	379.40	1.32	500.00
Lactococcus lactis	*Firmicutes*	*Bacilli*	*Lactobacillales*	*Streptococcaceae*	*Lactococcus*	587.40	0.85	500.00
Lactobacillus rhamnosus GG	*Firmicutes*	*Bacilli*	*Lactobacillales*	*Lactobacillaceae*	*Lactobacillus*	595.00	0.84	500.00
Staphylococcus aureus	*Firmicutes*	*Bacilli*	*Bacillales*	*Staphylococcaceae*	*Staphylococcus*	101.20	4.94	500.00
Erysipelothrix rhusiopathiae	*Firmicutes*	*Erysipelotrichi*	*Erysipelotrichales*	*Erysipelotrichaceae*	*Erysipelothrix*	30.80	16.23	500.00
Haemopilus parasuis	*Proteobacteria*	*Gammaproteobacteria*	*Pasteurellales*	*Pasteurellaceae*	*Haemophilus*	44.00	11.36	500.00
Staphylococcus .sp	*Proteobacteria*	*Gammaproteobacteria*	*Pasteurellales*	*Pasteurellaceae*	*Pasteurella*	254.10	1.97	500.00
Proteus mirabilis	*Proteobacteria*	*Gammaproteobacteria*	*Enterobacteriale*	*Enterobacteriaceae*	*Proteus*	132.80	3.77	500.00
Enteropathogenic E.coli	*Proteobacteria*	*Gammaproteobacteria*	*Enterobacteriale*	*Enterobacteriaceae*	*Escherichia*	632.60	0.79	500.00
Pseudomonas aeruginosa	*Proteobacteria*	*Gamma proteobacteria*	*Pseudomonadales*	*Pseudomonadaceae*	*Pseudomonas*	399.90	1.25	500.00
Microbacterium lacticum	*Actinobacteria*	*Actinobacteria*	*Actinomycetales*	*Microbacteriaceae*	*Microbacterium*	126.20	3.96	500.00
Trueperella pyogenes	*Actinobacteria*	*Actinobacteria*	*Actinomycetales*	*Actinomycetaceae*	*Trueperella*	51.00	9.80	500.00

b. Conformity of MC between qPCR and MiSeq quantitations.

Bacterial species	Genus	Raw reads of Mock	Clean reads of Mock	Assigned reads of Mock	Reads assigned at the genus level	qPCR		MiSeq	MiSeq/ qPCR
						Copies in mixed mock DNA	Percent	Percent	
Bacillus natto	Bacillus	55,102	52,516	47,512	22,137	4.91E+09	38.84%	50.50%	1.30
Bacillus subtilis						3.71E+09			
Bacillus licheniformis						3.13E+09			
Bacillus cereus						9.04E+07			
Clostridium perfringens	Clostridium				5,032	2.96E+09	9.72%	11.48%	1.18
Lactococcus lactis	Lactococcus				2,023	1.27E+09	4.16%	4.62%	1.11
Lactobacillus rhamnosus GG	Lactobacillus				513	5.85E+08	1.92%	1.17%	0.61
Staphylococcus aureus	Staphylococcus				1,142	7.92E+08	2.60%	2.61%	1.00
Erysipelothrix rhusiopathiae	Erysipelothrix				1,581	1.19E+09	3.92%	3.61%	0.92
Haemopilus parasuis	Haemophilus				1,316	3.21E+09	10.54%	3.00%	0.28
Staphylococcus .sp	Pasteurella				776	7.65E+08	2.51%	1.77%	0.71
Proteus mirabilis	Proteus				1,540	3.12E+09	10.25%	3.51%	0.34
Enteropathogenic E.coli	Escherichia				415	4.69E+08	1.54%	0.95%	0.61
Pseudomonas aeruginosa	Pseudomonas				1,597	1.37E+09	4.48%	3.64%	0.81
Microbacterium lacticum	Microbacterium				4,300	2.08E+09	6.83%	9.81%	1.44
Trueperella pyogenes	Trueperella				1,460	8.20E+08	2.69%	3.33%	1.24

Table S2. Summary of study samples and of fecal bacteria 16S rRNA amplicon sequence datasets

SampleID	Gender	Family	Mother	Phenotype	Sample Source	Time Sampling	clean reads	Barcode Sequence	Reads assigned otu	No. of otu
HS1	M	sow	–	Healthy	Feces	5 days after delivery	24892	TCCCTTGTCTCC	19037	970
HS2	M	sow	–	Healthy	Feces	5 days after delivery	36824	ACGAGACTGATT	29365	1455
HS3	M	sow	–	Healthy	Feces	5 days after delivery	32373	GCTGTACGGATT	25256	1561
AS1	M	sow	–	Asymptomatic	Feces	6 days after delivery	25193	ATCACCAGGTGT	20813	1004
AS2	M	sow	–	Asymptomatic	Feces	8 days after delivery	20241	TGGTCAACGATA	17121	768
AS3	M	sow	–	Asymptomatic	Feces	7 days after delivery	62117	ATCGCACAGTAA	49927	1183
AS4	M	sow	–	Asymptomatic	Feces	8 days after delivery	36048	GTCGTGTAGCCT	30697	1034
DS1	M	sow	–	Diarrheal	Feces	7 days after delivery	65244	ACCGGTATGTAC	57791	637
DS2	M	sow	–	Diarrheal	Feces	4 days after delivery	41630	AATTGTGTCGGA	36777	948
DS3	M	sow	–	Diarrheal	Feces	7 days after delivery	23870	TGCATACACTGG	19723	907
DS4	M	sow	–	Diarrheal	Feces	7 days after delivery	24367	AGTCGAACGAGG	20592	790
HP1	F	piglet	HS1	Healthy	Feces & Intestinal contents	5d	52425	ACCAGTGACTCA	47180	622
HP2	M	piglet	HS1	Healthy	Feces & Intestinal contents	5d	51565	GAATACCAAGTC	45238	730
HP3	F	piglet	HS2	Healthy	Feces & Intestinal contents	5d	26482	GTAGATCGTGTA	24109	364
HP4	M	piglet	HS2	Healthy	Feces & Intestinal contents	5d	38063	TAACGTGTGTGC	35444	215
AP1	F	piglet	DS1	Asymptomatic	Feces & Intestinal contents	7d	39861	CATTATGGCGTG	35106	494
AP2	M	piglet	DS1	Asymptomatic	Feces & Intestinal contents	6d	42423	CCAATACGCCTG	39339	315
AP3	F	piglet	DS2	Asymptomatic	Feces & Intestinal contents	3d	87496	GATCTGCGATCC	77746	565

AP4	M	piglet	DS2	Asymptomatic	Feces & Intestinal contents	4d	31337	CAGCTCATCAGC	27991	590
AP5	M	piglet	DS3	Asymptomatic	Feces & Intestinal contents	7d	59401	CAAACAACAGCT	54802	342
AP6	F	piglet	DS3	Asymptomatic	Feces & Intestinal contents	7d	46010	GCAACACCATCC	42403	291
AP7	F	piglet	DS4	Asymptomatic	Feces & Intestinal contents	8d	27836	GCGATATATCGC	24570	371
AP8	M	piglet	AS1	Asymptomatic	Feces & Intestinal contents	8d	29898	CGAGCAATCCTA	25217	788
AP9	F	piglet	AS1	Asymptomatic	Feces & Intestinal contents	9d	49315	AGTCGTGCACAT	44587	445
AP10	M	piglet	AS2	Asymptomatic	Feces & Intestinal contents	3d	32324	GTATCTGCGCGT	29137	294
AP11	F	piglet	AS2	Asymptomatic	Feces & Intestinal contents	3d	27284	CGAGGGAAAGTC	22478	233
AP12	F	piglet	AS3	Asymptomatic	Feces & Intestinal contents	7d	34535	CAAATTCGGGAT	30989	509
AP13	M	piglet	AS4	Asymptomatic	Feces & Intestinal contents	8d	37624	AGATTGACCAAC	31992	566
AP14	F	piglet	AS4	Asymptomatic	Feces & Intestinal contents	8d	26618	AGTTACGAGCTA	21616	820
AP15	M	piglet	AS4	Asymptomatic	Feces & Intestinal contents	8d	36829	GCATATGCACTG	29792	700
DP1	F	piglet	DS1	Diarrheal	Feces & Intestinal contents	8d	21720	CAACTCCCGTGA	19347	308
DP2	M	piglet	DS1	Diarrheal	Feces & Intestinal contents	8d	39469	TTGCGTTAGCAG	36292	262
DP3	F	piglet	DS2	Diarrheal	Feces & Intestinal contents	3d	42777	TACGAGCCCTAA	39183	560
DP4	M	piglet	DS2	Diarrheal	Feces & Intestinal contents	4d	24678	CACTACGCTAGA	21633	320

DP5	F	piglet	DS3	Diarrheal	Feces & Intestinal contents	7d	36651	TGCAGTCCTCGA	32732	394
DP6	M	piglet	DS3	Diarrheal	Feces & Intestinal contents	7d	29452	ACCATAGCTCCG	26038	560
DP7	F	piglet	DS4	Diarrheal	Feces & Intestinal contents	5d	33382	TCGACATCTCTT	30892	275
DP8	F	piglet	DS4	Diarrheal	Feces & Intestinal contents	5d	25983	GAACACTTTGGA	22430	497
DP9	M	piglet	AS1	Diarrheal	Feces & Intestinal contents	7d	46236	GAGCCATCTGTA	41307	540
DP10	F	piglet	AS1	Diarrheal	Feces & Intestinal contents	7d	31416	TTGGGTACACGT	29414	143
DP11	M	piglet	AS2	Diarrheal	Feces & Intestinal contents	3d	22636	AAGGCGCTCCTT	19595	246
DP12	F	piglet	AS2	Diarrheal	Feces & Intestinal contents	3d	47984	TAATACGGATCG	42449	327
DP13	M	piglet	AS3	Diarrheal	Feces & Intestinal contents	7d	32571	TCGGAATTAGAC	30487	294
DP14	F	piglet	AS3	Diarrheal	Feces & Intestinal contents	7d	39791	TGTGAATTCGGA	35657	390
DP15	M	piglet	AS4	Diarrheal	Feces & Intestinal contents	8d	29271	CATTCGTGGCGT	23855	576

Table S3 Statistics of alpha diversity indices

Group	SampleID	Shannon	Chao1	Good's coverage	Observed species	Simpson
HS	HS1	6.68	1467	0.96	970	0.95
	HS2	7.40	3189	0.96	1455	0.98
	HS3	7.64	2597	0.96	1561	0.98
AS	AS1	7.65	2853	0.95	1004	0.99
	AS2	7.09	1991	0.96	768	0.98
	AS3	7.69	2699	0.97	1183	0.99
	AS4	6.13	2366	0.97	1034	0.91
DS	DS1	4.96	2006	0.99	637	0.83
	DS2	7.19	2152	0.98	948	0.98
	DS3	7.07	1923	0.96	907	0.98
	DS4	6.69	1902	0.97	790	0.97
HP	HP1	5.50	1738	0.98	622	0.94
	HP2	5.53	2128	0.98	730	0.90
	HP3	4.62	1054	0.98	364	0.90
	HP4	2.94	598	0.99	215	0.74
AP	AP1	3.71	1842	0.98	494	0.83
	AP2	3.46	786	0.99	315	0.80
	AP3	5.50	2294	0.98	565	0.94
	AP4	5.11	1579	0.98	590	0.91
	AP5	3.66	1309	0.99	342	0.76
	AP6	3.14	1151	0.99	291	0.68
	AP7	4.53	1462	0.98	371	0.92
	AP8	5.65	2460	0.96	788	0.92
	AP9	3.57	1948	0.98	445	0.65
	AP10	4.11	629	0.99	294	0.89
	AP11	4.27	682	0.99	233	0.89
	AP12	4.77	1351	0.98	509	0.90
	AP13	6.53	2018	0.97	566	0.97
	AP14	6.65	2585	0.96	820	0.96
	AP15	6.85	2779	0.96	700	0.97
DP	DP1	3.82	1180	0.98	308	0.84
	DP2	2.70	1427	0.99	262	0.59
	DP3	4.49	1689	0.98	560	0.84
	DP4	3.92	1334	0.98	320	0.86
	DP5	4.20	1836	0.98	394	0.87
	DP6	6.11	1833	0.97	560	0.97
	DP7	3.64	716	0.99	275	0.83
	DP8	4.90	1548	0.97	497	0.91
	DP9	4.37	2442	0.98	540	0.77
	DP10	1.71	429	0.99	143	0.42
	DP11	4.02	1346	0.98	246	0.90
	DP12	3.35	1581	0.98	327	0.77
	DP13	3.62	726	0.99	294	0.82
	DP14	5.04	991	0.99	390	0.93
	DP15	6.50	2779	0.95	576	0.96

Table S4 Comparison of bacteria abundance between HS and HP at phylum and genus level

Taxon	HP	HS
a. phylum level		
Bacteroidetes	76.2902%	47.3852%
Firmicutes	21.1751%	34.5693%
Proteobacteria	2.1175%	5.7448%
Spirochaetes	0.0224%	4.6424%
Tenericutes	0.0316%	4.0518%
Verrucomicrobia	0.0000%	1.3600%
Fibrobacteres	0.0000%	0.9164%
Planctomycetes	0.0000%	0.3825%
Cyanobacteria	0.0000%	0.1322%
Actinobacteria	0.0461%	0.1145%
Lentisphaerae	0.0165%	0.0940%
Thermi	0.0020%	0.0000%
Fusobacteria	0.1915%	0.0441%
Synergistetes	0.0000%	0.0091%
Unclassified	0.1073%	0.5536%
b. genus level		
Bacteroides	49.3542%	1.3875%
Prevotella	12.2826%	9.5658%
Parabacteroides	8.6852%	1.6454%
Lactobacillus	8.2114%	0.8111%
Oscillospira	1.2851%	2.5415%
YRC22	0.0599%	7.0746%
CF231	0.0671%	1.7188%
Streptococcus	0.6679%	2.3392%
Ruminococcus	0.4619%	1.1133%
Treponema	0.0112%	4.0946%
Other genera	3.6250%	7.5994%
Unclassified	15.2884%	60.1089%

Table S5 The bacterial abundance at the phylum level were comparison by

ANOVA among the sow (a) and piglet (b) groups

a. sow

Microbiota	Abundance (%)		
	HS	AS	DS
Firmicutes	34.57 ± 5.50 [Ab]	54.36 ± 9.74 [Aa]	35.30 ± 7.45 [Ab]
Bacteroidetes	47.39 ± 7.42 [Aa]	33.69 ± 7.86 [Aa]	42.35 ± 13.02 [Aa]
Actinobacteria	0.11 ± 0.05 [ABb]	0.28 ± 0.11 [Aa]	0.05 ± 0.09 [Bb]
Proteobacteria	5.74 ± 4.29 [Aa]	2.16 ± 0.96 [Aab]	1.26 ± 0.60 [Ab]
Spirochaetes	4.64 ± 2.74 [Aa]	5.27 ± 0.83 [Aa]	9.31 ± 5.47 [Aa]
Fibrobacteres	0.92 ± 0.66 [Aab]	1.16 ± 0.71 [Aa]	0.01 ± 0.03 [Ab]
Tenericutes	4.05 ± 3.09 [Aa]	1.89 ± 1.71 [Aa]	8.05 ± 5.29 [Aa]
Verrucomicrobia	1.36 ± 0.97 [Aa]	0.58 ± 0.42 [Aa]	0.11 ± 0.14 [Aa]
Fusobacteria	0.04 ± 0.07 [Aa]	0.04 ± 0.08 [Aa]	0.37 ± 0.42 [Aa]
Cyanobacteria	0.13 ± 0.21 [Aa]	0.04 ± 0.03 [Aa]	0.02 ± 0.02 [Aa]
TM7	0.00 ± 0.00 [Aa]	0.01 ± 0.02 [Aa]	2.41 ± 4.52 [Aa]
Others/unclassified	1.04 ± 0.80 [Aa]	0.51 ± 0.47 [Aa]	0.77 ± 0.58 [Aa]

b. piglet

Microbiota	Abundance (%)		
	HS	AS	DS
Firmicutes	19.06 ± 15.20 [Aa]	44.32 ± 29.55 [Aa]	49.65 ± 29.13 [Aa]
Bacteroidetes	78.80 ± 17.33 [Aa]	23.05 ± 20.15 [Bb]	21.09 ± 23.11 [Bb]
Proteobacteria	1.77 ± 1.98 [Aa]	16.43 ± 27.34 [Aa]	12.74 ± 15.87 [Aa]
Fusobacteria	0.17 ± 0.15 [Aa]	15.36 ± 25.59 [Aa]	14.23 ± 24.65 [Aa]
Actinobacteria	0.04 ± 0.04 [Aa]	0.09 ± 0.13 [Aa]	1.83 ± 7.01 [Aa]
Tenericutes	0.03 ± 0.03 [ABab]	0.10 ± 0.09 [Bb]	0.03 ± 0.04 [Aa]
Spirochaetes	0.02 ± 0.02 [Aa]	0.05 ± 0.06 [Aa]	0.03 ± 0.07 [Aa]
Fibrobacteres	0.00 ± 0.00 [Aa]	0.00 ± 0.00 [Aa]	0.00 ± 0.00 [Aa]
Verrucomicrobia	0.00 ± 0.00 [Aa]	0.04 ± 0.12 [Aa]	0.06 ± 0.20 [Aa]
Cyanobacteria	0.00 ± 0.00 [Aa]	0.00 ± 0.00 [Aa]	0.00 ± 0.00 [Aa]
TM7	0.00 ± 0.00 [Aa]	0.03 ± 0.06 [Aa]	0.00 ± 0.00 [Aa]
Others/unclassified	0.11 ± 0.10 [Aa]	0.53 ± 1.10 [Aa]	0.34 ± 0.86 [Aa]

Table S6 Relative differences in microbial taxa abundance for (a) HS versus (vs.) AS vs. DS, (b) HS vs. AS, (c) HS vs. DS, (d) AS vs. DS, (e) HP vs. AP vs. DP, (f) HP vs. AP, (g) HP vs. DP, and (h) AP vs. DP.

a. HS vs. AS vs. DS

Phylum	Class	Order	Family	Genus	Species	LDA Score	Riched group	p-value
						5.735	AS	0.048*
	Clostridia	*Clostridiales*	*Mogibacteriaceae*			3.729	AS	0.041*
	Clostridia	*Clostridiales*	*Other*			4.703	AS	0.029*
	Clostridia	*Clostridiales*	*Christensenellaceae*			4.665	AS	0.024*
	Clostridia	*Clostridiales*	*Ruminococcaceae*	*Oscillospira*		4.503	AS	0.027*
Firmicutes	*Bacilli*	*Lactobacillales*	*Streptococcaceae*	*Streptococcus*		2.958	AS	0.020*
	Erysipelotrichi	*Erysipelotrichales*	*Erysipelotrichaceae*	*L7A_E11*		3.789	DS	0.040*
	Clostridia	*Clostridiales*	*Clostridiaceae*	*02d06*		3.770	HS	0.03*
	Clostridia	*Clostridiales*	*Veillonellaceae*	*Anaerovibrio*		2.764	HS	0.028*
	Clostridia	*Clostridiales*	*Clostridiaceae*	*Clostridium*	*butyricum*	3.445	HS	0.028*
	Bacilli	*Lactobacillales*	*Lactobacillaceae*	*Lactobacillus*	*salivarius*	3.025	DS	0.030*
	Flavobacteriia					2.926	HS	0.016*
	Bacteroidia	*Bacteroidales*				5.110	HS	0.049*
	Flavobacteriia	*Flavobacteriales*				2.926	HS	0.016*
	Flavobacteriia	*Flavobacteriales*	*Flavobacteriaceae*			2.805	HS	0.016*
Bacteroidetes	*Bacteroidia*	*Bacteroidales*	*S24_7*			4.865	AS	0.028*
	Bacteroidia	*Bacteroidales*	*RF16*			3.632	DS	0.048*
	Bacteroidia	*Bacteroidales*	*Prevotellaceae*	Other		2.477	HS	0.016*
	Bacteroidia	*Bacteroidales*	*Prevotellaceae*	*Prevotella*	Other	3.095	HS	0.039*
	Bacteroidia	*Bacteroidales*	*Paraprevotellaceae*	*YRC22*		4.938	HS	0.019*

Proteobacteria	*Gammaproteobacteria*				4.666	HS	0.020*
	Gammaproteobacteria	*Xanthomonadales*			3.156	HS	0.016*
	Deltaproteobacteria	*Desulfovibrionales*			3.674	AS	0.035*
	Gammaproteobacteria	*Aeromonadales*			2.851	HS	0.046*
	Gammaproteobacteria	*Pseudomonadales*			4.501	HS	0.036*
	Gammaproteobacteria	*Xanthomonadales*	*Xanthomonadaceae*		3.156	HS	0.016*
	Gammaproteobacteria	*Xanthomonadales*	*Xanthomonadaceae*	*Stenotrophomonas*	2.584	HS	0.016*
	Deltaproteobacteria	*Desulfovibrionales*	*Desulfovibrionaceae*	*Desulfovibrio*	3.655	AS	0.035*
Actinobacteria					3.447	AS	0.022*
	Coriobacteriia				3.447	AS	0.009**
	Coriobacteriia	*Coriobacteriales*			3.447	AS	0.009**
	Coriobacteriia	*Coriobacteriales*	*Coriobacteriaceae*	*Other*	3.366	AS	0.009**
Fibrobacteres					4.064	AS	0.028*
	Fibrobacteria				4.064	AS	0.028*
	Fibrobacteria	*Fibrobacterales*			4.064	AS	0.028*
	Fibrobacteria	*Fibrobacterales*	*Fibrobacteraceae*		4.064	AS	0.028*
	Fibrobacteria	*Fibrobacterales*	*Fibrobacteraceae*	*Fibrobacter*	4.064	AS	0.025*
Tenericutes	*RF3*				4.004	DS	0.02*
	RF3	*ML615J_28*			4.004	DS	0.02*
	Mollicutes	*Anaeroplasmatales*			4.466	DS	0.031*
	Mollicutes	*Anaeroplasmatales*	*Anaeroplasmataceae*	*Anaeroplasma*	4.402	DS	0.031*

*: $p<0.05$. **: $p<0.01$.

b. HS vs. DS

Phylum	Class	Order	Family	Genus	Species	LDA Score	Riched group	p-value
Firmicutes	*Clostridia*	*Clostridiales*	*Christensenellaceae*			4.388	HS	0.034*
	Clostridia	*Clostridiales*	*Mogibacteriaceae*			3.600	DS	0.034*
	Clostridia	*Clostridiales*	*Peptostreptococcaceae*			3.848	DS	0.032*
	Clostridia	*Clostridiales*	*Peptostreptococcaceae*	*Peptostreptococcus*		3.421	DS	0.028*
	Clostridia	*Clostridiales*	*Ruminococcaceae*	*Oscillospira*		4.385	HS	0.034*
	Clostridia	*Clostridiales*	*Clostridiaceae*	*02d06*		3.770	HS	0.032*
	Erysipelotrichi	*Erysipelotrichales*	*Erysipelotrichaceae*	*L7A_E11*		3.789	DS	0.034*
	Bacilli	*Lactobacillales*	*Streptococcaceae*	*Streptococcus*		2.724	HS	0.019*
	Clostridia	*Clostridiales*	*Veillonellaceae*	*Anaerovibrio*		2.764	HS	0.019*
	Clostridia	*Clostridiales*	*Clostridiaceae*	*Clostridium*	*butyricum*	3.445	HS	0.028*
	Clostridia	*Clostridiales*	*Lachnospiraceae*	*Ruminococcus*	*gnavus*	2.990	DS	0.034*
Bacteroidetes	*Flavobacteriia*					2.926	HS	0.028*
	Bacteroidia	*Bacteroidales*				5.110	HS	0.034*
	Flavobacteriia	*Flavobacteriales*				2.926	HS	0.028*
	Flavobacteriia	*Flavobacteriales*	*Flavobacteriaceae*			2.805	HS	0.028*
	Flavobacteriia	*Flavobacteriales*	*Cryomorphaceae*			2.315	HS	0.019*
	Bacteroidia	*Bacteroidales*	*BS11*			3.630	HS	0.019*
	Bacteroidia	*Bacteroidales*	*Porphyromonadaceae*			4.197	HS	0.034*
	Bacteroidia	*Bacteroidales*	*Paraprevotellaceae*	*YRC22*		4.938	HS	0.032*
	Flavobacteriia	*Flavobacteriales*	*Cryomorphaceae*	*Fluviicola*		2.315	HS	0.019*
	Bacteroidia	*Bacteroidales*	*Porphyromonadaceae*	*Parabacteroides*	*distasonis*	3.352	HS	0.034*
	Bacteroidia	*Bacteroidales*	*Prevotellaceae*	*Prevotellaceae*	Other	2.477	HS	0.028*
	Bacteroidia	*Bacteroidales*	*Prevotellaceae*	*Prevotella*	Other	3.095	HS	0.028*

Proteobacteria						4.759	HS	0.034*
	Gammaproteobacteria					4.666	HS	0.034*
	Gammaproteobacteria	*Xanthomonadales*				3.156	HS	0.028*
	Gammaproteobacteria	*Pseudomonadales*				4.501	HS	0.028*
	Alphaproteobacteria	*Rhodobacterales*				3.193	HS	0.019*
	Gammaproteobacteria	*Xanthomonadales*	*Xanthomonadaceae*			3.156	HS	0.028*
	Betaproteobacteria	*Burkholderiales*	*Comamonadaceae*			3.016	HS	0.019*
	Alphaproteobacteria	*Rhodobacterales*	*Rhodobacteraceae*			3.193	HS	0.019*
	Gammaproteobacteria	*Xanthomonadales*	*Xanthomonadaceae*	*Stenotrophomonas*		2.584	HS	0.028*
	Betaproteobacteria	*Burkholderiales*	*Comamonadaceae*	*Comamonas*		2.576	HS	0.019*
	Alphaproteobacteria	*Rhodobacterales*	*Rhodobacteraceae*	*Rhodobacter*		3.193	HS	0.019*
	Betaproteobacteria	*Burkholderiales*	*Comamonadaceae*	*Other*		2.313	HS	0.019*
	Betaproteobacteria	*Burkholderiales*	*Comamonadaceae*	*Leptothrix*		2.279	HS	0.019*
	Gammaproteobacteria	*Pseudomonadales*	*Pseudomonadaceae*	*Pseudomonas*	*Other*	2.706	HS	0.019*
	Gammaproteobacteria	*Pseudomonadales*	*Pseudomonadaceae*	*Pseudomonas*	*viridiflava*	2.804	HS	0.019*
Actinobacteria	*Coriobacteriia*					2.933	HS	0.019*
	Coriobacteriia	*Coriobacteriales*				2.933	HS	0.019*
	Actinobacteria	*Actinomycetales*	*Propionibacteriaceae*			2.459	HS	0.019*
	Coriobacteriia	*Coriobacteriales*	*Coriobacteriaceae*			2.351	HS	0.019*
	Actinobacteria	*Actinomycetales*	*Propionibacteriaceae*	*Propionibacterium*		2.459	HS	0.019*
	Coriobacteriia	*Coriobacteriales*	*Coriobacteriaceae*	*Other*		2.801	HS	0.019*
	Actinobacteria	*Actinomycetales*	*Propionibacteriaceae*	*Propionibacterium*	*acnes*	2.459	HS	0.019*
Fibrobacteres						3.962	HS	0.028*
	Fibrobacteria					3.962	HS	0.028*
	Fibrobacteria	*Fibrobacterales*				3.962	HS	0.028*

	Fibrobacteria	*Fibrobacterales*	*Fibrobacteraceae*			3.962	HS	0.028*
	Fibrobacteria	*Fibrobacterales*	*Fibrobacteraceae*	*Fibrobacter*		3.753	HS	0.028*
Tenericutes	*RF3*					4.004	DS	0.034*
	Mollicutes	*Anaeroplasmatales*				4.466	DS	0.034*
	RF3	*ML615J_28*				4.004	DS	0.034*
	Mollicutes	*Anaeroplasmatales*	*Anaeroplasmataceae*	*Anaeroplasma*		4.402	DS	0.034*

c. HS vs. AS

Phylum	Class	Order	Family	Genus	Species	LDA Score	Riched group	p-value
Firmicutes						5.735	AS	0.034*
	Clostridia	*Clostridiales*	*Mogibacteriaceae*			3.729	AS	0.034*
	Clostridia	*Clostridiales*	*Other*			4.703	AS	0.034*
	Clostridia	*Clostridiales*	*Clostridiaceae*	*Clostridium*	*butyricum*	3.445	HS	0.028*
Bacteroidetes						5.676	HS	0.034*
	Bacteroidia					5.674	HS	0.034*
	Flavobacteriia					2.926	HS	0.019*
	Bacteroidia	*Bacteroidales*				5.110	HS	0.034*
	Flavobacteriia	*Flavobacteriales*				2.926	HS	0.019*
	Flavobacteriia	*Flavobacteriales*	*Flavobacteriaceae*			2.805	HS	0.019*
	Flavobacteriia	*Flavobacteriales*	*Cryomorphaceae*			2.315	HS	0.019*
	Bacteroidia	*Bacteroidales*	*BS11*			3.630	HS	0.019*
	Flavobacteriia	*Flavobacteriales*	*Cryomorphaceae*	*Fluviicola*		2.315	HS	0.019*
	Bacteroidia	*Bacteroidales*	*Prevotellaceae*	*Prevotellaceae*	*Other*	2.477	HS	0.019*
	Alphaproteobacteria					3.295	HS	0.028*

	Gammaproteobacteria	*Pseudomonadales*				4.501	HS	0.032*
	Gammaproteobacteria	*Xanthomonadales*				3.156	HS	0.019*
Proteobacteria	*Alphaproteobacteria*	*Rhodobacterales*				3.193	HS	0.019*
	Gammaproteobacteria	*Aeromonadales*				2.851	HS	0.019*
	Alphaproteobacteria	*Rhodobacterales*	*Rhodobacteraceae*			3.193	HS	0.019*
	Gammaproteobacteria	*Xanthomonadales*	*Xanthomonadaceae*			3.156	HS	0.019*
	Betaproteobacteria	*Burkholderiales*	*Comamonadaceae*			3.016	HS	0.019*
	Betaproteobacteria	*Burkholderiales*	*Comamonadaceae*	*Comamonas*		2.576	HS	0.019*
	Betaproteobacteria	*Burkholderiales*	*Comamonadaceae*	*Leptothrix*		2.279	HS	0.019*
	Gammaproteobacteria	*Xanthomonadales*	*Xanthomonadaceae*	*Stenotrophomonas*		2.584	HS	0.019*
	Gammaproteobacteria	*Pseudomonadales*	*Pseudomonadaceae*	*Pseudomonas*		2.804	HS	0.019*
	Alphaproteobacteria	*Rhodobacterales*	*Rhodobacteraceae*	*Rhodobacter*		3.193	HS	0.019*
	Betaproteobacteria	*Burkholderiales*	*Comamonadaceae*	*Comamonadaceae*	*Other*	2.313	HS	0.019*
	Gammaproteobacteria	*Pseudomonadales*	*Pseudomonadaceae*	*Pseudomonas*	*Other*	2.706	HS	0.019*
						3.447	AS	0.034*
	Actinobacteria					2.459	HS	0.019*
	Coriobacteriia					3.447	AS	0.034*
	Actinobacteria	*Actinomycetales*				2.459	HS	0.019*
Actinobacteria	*Coriobacteriia*	*Coriobacteriales*				3.447	AS	0.034*
	Actinobacteria	*Actinomycetales*	*Propionibacteriaceae*			2.459	HS	0.019*
	Actinobacteria	*Actinomycetales*	*Propionibacteriaceae*	*Propionibacterium*		2.459	HS	0.019*
	Coriobacteriia	*Coriobacteriales*	*Coriobacteriaceae*	*Coriobacteriaceae*	*Other*	3.366	AS	0.034*
	Actinobacteria	*Actinomycetales*	*Propionibacteriaceae*	*Propionibacterium*	*acnes*	2.459	HS	0.019*

d. AS vs. DS

Phylum	Class	Order	Family	Genus	LDA Score	Riched group	p-value
					5.735	AS	0.043*
	Clostridia				5.624	AS	0.043*
	Clostridia	*Clostridiales*	*Clostridiales*		4.703	AS	0.021*
Firmicutes	*Clostridia*	*Clostridiales*	*Christensenellaceae*		4.665	AS	0.021*
	Clostridia	*Clostridiales*	*Ruminococcaceae*	*Oscillospira*	4.503	AS	0.021*
	Clostridia	*Clostridiales*	*Clostridiaceae*	*02d06*	3.612	AS	0.020*
	Bacteroidia	*Bacteroidales*	*RF16*		3.632	DS	0.021*
	Bacteroidia	*Bacteroidales*	*S24_7*		4.865	AS	0.021*
	Bacteroidia	*Bacteroidales*	*Bacteroidaceae*		4.494	DS	0.043*
Bacteroidetes	*Bacteroidia*	*Bacteroidales*	*Bacteroidaceae*	*Bacteroides*	4.494	DS	0.043*
	Bacteroidia	*Bacteroidales*	*Paraprevotellaceae*	*YRC22*	3.835	AS	0.020*
	Bacteroidia	*Bacteroidales*	*Rikenellaceae*	*Other*	2.141	DS	0.047*
	Deltaproteobacteria				3.751	AS	0.021*
	Gammaproteobacteria				4.131	AS	0.021*
	Deltaproteobacteria	*Desulfovibrionales*			3.674	AS	0.021*
Proteobacteria	*Gammaproteobacteria*	*Enterobacteriales*			4.126	AS	0.021*
	Gammaproteobacteria	*Enterobacteriales*	*Enterobacteriaceae*		3.386	AS	0.021*
	Deltaproteobacteria	*Desulfovibrionales*	*Desulfovibrionaceae*		3.674	AS	0.021*
	Deltaproteobacteria	*Desulfovibrionales*	*Desulfovibrionaceae*	*Desulfovibrio*	3.655	AS	0.021*
	Gammaproteobacteria	*Enterobacteriales*	*Enterobacteriaceae*	*Other*	4.037	AS	0.021*
Actinobacteria					3.447	AS	0.018*
	Coriobacteriia				3.447	AS	0.014*

	Coriobacteriia	*Coriobacteriales*			3.447	AS	0.014*
	Coriobacteriia	*Coriobacteriales*	*Coriobacteriaceae*		2.461	AS	0.047*
	Coriobacteriia	*Coriobacteriales*	*Coriobacteriaceae*	*Other*	3.366	AS	0.014*
Fibrobacteres					4.064	AS	0.018*
	Fibrobacteria				4.064	AS	0.018*
	Fibrobacteria	*Fibrobacterales*			4.064	AS	0.018*
	Fibrobacteria	*Fibrobacterales*	*Fibrobacteraceae*		4.064	AS	0.018*
	Fibrobacteria	*Fibrobacterales*	*Fibrobacteraceae*	*Fibrobacter*	4.064	AS	0.018*
Tenericutes					4.906	DS	0.043*
	RF3				4.004	DS	0.021*
	RF3	*ML615J_28*			4.004	DS	0.021*
Verrucomicrobia					3.765	AS	0.043*
	Verruco_5				3.753	AS	0.042*
	Verruco_5	*WCHB1_41*			3.753	AS	0.042*
	Verruco_5	*WCHB1_41*	*RFP12*		3.742	AS	0.042*

e. HP vs. AP vs. DP

Phylum	Class	Order	Family	Genus	LDA Score	Riched group	p-value
Firmicutes	*Bacilli*	*Lactobacillales*	*Enterococcaceae*		3.287	DP	0.039*
	Clostridia	*Clostridiales*	*Veillonellaceae*		4.846	DP	0.021*
	Clostridia	*Clostridiales*	*Clostridiaceae*	*SMB53*	3.997	AP	0.016*
	Clostridia	*Clostridiales*	*Peptostreptococcaceae*	*Peptostreptococcus*	4.165	DP	0.042*
	Bacilli	*Lactobacillales*	*Enterococcaceae*	*Enterococcus*	3.209	DP	0.039*
	Bacilli	*Lactobacillales*	*Lactobacillaceae*	*Lactobacillus*	4.828	DP	0.012*
Bacteroidetes	*Bacteroidia*	*Bacteroidales*			4.038	AP	0.020*
	Bacteroidia	*Bacteroidales*	*Bacteroidales*		3.707	HP	0.011*
	Bacteroidia	*Bacteroidales*	*Prevotellaceae*	*Prevotella*	4.962	HP	0.041*
	Bacteroidia	*Bacteroidales*	*Prevotellaceae*	*Prevotella_others*	3.690	HP	0.032*
	Bacteroidia	*Bacteroidales*	*Odoribacteraceae*	*Odoribacter*	3.172	HP	0.026*
	Bacteroidia	*Bacteroidales*	*Porphyromonadaceae*	*Parabacteroides*	3.241	HP	0.007**
	Bacteroidia	*Bacteroidales*	*Paraprevotellaceae*	*CF231*	2.887	AP	0.031*
	Bacteroidia	*Bacteroidales*	*Porphyromonadaceae*	*Porphyromonas*	3.284	DP	0.006**
Proteobacteria	*Alphaproteobacteria*				2.526	AP	0.047*
	Betaproteobacteria				4.424	AP	0.026*
	Betaproteobacteria	*Burkholderiales*			4.421	AP	0.024*
	Gammaproteobacteria	*Xanthomonadales*			1.774	HP	0.041*
	Gammaproteobacteria	*Xanthomonadales*	*Xanthomonadaceae*		1.774	HP	0.041*
	Gammaproteobacteria	*Xanthomonadales*	*Xanthomonadaceae*	*Stenotrophomonas*	1.774	HP	0.041*
	Gammaproteobacteria	*Pasteurellales*	*Pasteurellaceae*	*Other*	3.262	HP	0.028*
Tenericutes	*Mollicutes*	*RF39*			2.569	AP	0.021*

f. HP vs. AP

Phylum	Class	Order	Family	Genus	Species	LDA Score	Riched group	p-value
Firmicutes	*Erysipelotrichi*	*Erysipelotrichales*	*Erysipelotrichaceae*	*Other*		2.490	AP	0.034*
	Clostridia	*Clostridiales*	*Clostridiaceae*	*Clostridium*	*butyricum*	1.580	HP	0.005**
	Clostridia	*Clostridiales*	*Clostridiaceae*	*Clostridium*	*perfringens*	3.553	AP	0.045*
Bacteroidetes						5.897	HP	0.005**
	Bacteroidia					5.896	HP	0.005**
	Flavobacteriia					2.111	HP	0.005**
	Flavobacteriia	*Flavobacteriales*				2.111	HP	0.005**
	Bacteroidia	*Bacteroidales*	*RF16*			2.350	HP	0.043*
	Flavobacteriia	*Flavobacteriales*	*Cryomorphaceae*			1.774	HP	0.005**
	Flavobacteriia	*Flavobacteriales*	*Flavobacteriaceae*			1.844	HP	0.005**
	Bacteroidia	*Bacteroidales*	*Bacteroidaceae*			5.741	HP	0.012*
	Bacteroidia	*Bacteroidales*	*Bacteroidaceae*	*Bacteroides*		5.213	HP	0.009**
	Flavobacteriia	*Flavobacteriales*	*Cryomorphaceae*	*Fluviicola*		1.774	HP	0.005**
	Flavobacteriia	*Flavobacteriales*	*Flavobacteriaceae*	*Flavobacterium*		1.844	HP	0.005**
	Bacteroidia	*Bacteroidales*	*Bacteroidaceae*	*Bacteroides*	*caccae*	3.149	HP	0.034*
	Bacteroidia	*Bacteroidales*	*Bacteroidaceae*	*Bacteroides*	*uniformis*	4.188	HP	0.016*
	Bacteroidia	*Bacteroidales*	*Bacteroidaceae*	*Bacteroides*	*ovatus*	4.873	HP	0.005**
Proteobacteria	*Alphaproteobacteria*	*Rhodobacterales*				2.422	HP	0.005**
	Alphaproteobacteria	*Rhodobacterales*	*Rhodobacteraceae*			2.422	HP	0.005**
	Betaproteobacteria	*Burkholderiales*	*Comamonadaceae*	*Leptothrix*		1.936	HP	0.005**

	Alphaproteobacteria	*Rhodobacterales*	*Rhodobacteraceae*	*Rhodobacter*		2.422	HP	0.005**
	Gammaproteobacteria	*Pseudomonadales*	*Pseudomonadaceae*	*Pseudomonas*	*viridiflava*	1.636	HP	0.005**
Actinobacteria	*Actinobacteria*	*Actinomycetales*	*Micrococcaceae*	*Rothia*		2.258	HP	0.041*
	Actinobacteria	*Actinomycetales*	*Micrococcaceae*	*Rothia*	*nasimurium*	2.258	HP	0.041*

g. HP vs. DP

Phylum	Class	Order	Family	Genus	Species	LDA Score	Riched group	p-value
Firmicutes						5.736	DP	0.028*
	Bacilli	*Lactobacillales*	*Enterococcaceae*			3.287	DP	0.034*
	Clostridia	*Clostridiales*	*Veillonellaceae*			4.846	DP	0.016*
	Bacilli	*Lactobacillales*	*Enterococcaceae*	*Enterococcus*		3.209	DP	0.034*
	Clostridia	*Clostridiales*	*Peptostreptococcaceae*	*Peptostreptococcus*		4.165	DP	0.026*
	Clostridia	*Clostridiales*	*Lachnospiraceae*	*Coprococcus*	*Other*	1.811	HP	0.041*
	Clostridia	*Clostridiales*	*Clostridiaceae*	*Clostridium*	*butyricum*	1.580	HP	0.005**
	Clostridia	*Clostridiales*	*Clostridiaceae*	*Clostridium*	*perfringens*	4.199	DP	0.035*
	Bacilli	*Lactobacillales*	*Lactobacillaceae*	*Lactobacillus*	*vaginalis*	4.828	DP	0.028*
Bacteroidetes						5.897	HP	0.004**
	Bacteroidia					5.896	HP	0.004**
	Bacteroidia	*Bacteroidales*				3.713	DP	0.007**
	Bacteroidia	*Bacteroidales*	*Bacteroidales*			3.707	HP	0.008**
	Bacteroidia	*Bacteroidales*	*RF16*			2.350	HP	0.005**
	Flavobacteriia	*Flavobacteriales*	*Flavobacteriaceae*			1.844	HP	0.005**
	Flavobacteriia	*Flavobacteriales*	*Cryomorphaceae*			1.774	HP	0.005**
	Bacteroidia	*Bacteroidales*	*Bacteroidaceae*			5.741	HP	0.036*

	Flavobacteriia	*Flavobacteriales*	*Flavobacteriaceae*	*Flavobacterium*		1.844	HP	0.005**
	Flavobacteriia	*Flavobacteriales*	*Cryomorphaceae*	*Fluviicola*		1.774	HP	0.005**
	Bacteroidia	*Bacteroidales*	*Paraprevotellaceae*	*CF231*		2.807	HP	0.009**
	Bacteroidia	*Bacteroidales*	*Bacteroidaceae*	*Bacteroides*		5.213	HP	0.035*
	Bacteroidia	*Bacteroidales*	*Odoribacteraceae*	*Odoribacter*		3.172	HP	0.021*
	Bacteroidia	*Bacteroidales*	*Prevotellaceae*	*Prevotella*		3.831	HP	0.020*
	Bacteroidia	*Bacteroidales*	*Porphyromonadaceae*	*Porphyromonas*		3.284	DP	0.022*
	Bacteroidia	*Bacteroidales*	*Prevotellaceae*	*Prevotella*	*Other*	3.690	HP	0.017*
	Bacteroidia	*Bacteroidales*	*Bacteroidaceae*	*Bacteroides*	*uniformis*	4.188	HP	0.012*
	Bacteroidia	*Bacteroidales*	*Prevotellaceae*	*Prevotella*	*stercorea*	4.962	HP	0.012*
	Bacteroidia	*Bacteroidales*	*Bacteroidaceae*	*Bacteroides*	*Other*	5.277	HP	0.045*
	Bacteroidia	*Bacteroidales*	*Prevotellaceae*	*Prevotella*	*copri*	2.691	HP	0.039*
	Bacteroidia	*Bacteroidales*	*Bacteroidaceae*	*Bacteroides*	*ovatus*	4.873	HP	0.003**
	Bacteroidia	*Bacteroidales*	*Porphyromonadaceae*	*Parabacteroides*	*Other*	3.241	HP	0.002**
	Bacteroidia	*Bacteroidales*	*Bacteroidaceae*	*Bacteroides*	*caccae*	3.149	HP	0.001**
	Alphaproteobacteria					2.422	HP	0.005**
	Gammaproteobacteria	*Xanthomonadales*				1.774	HP	0.005**
	Alphaproteobacteria	*Rhodobacterales*				2.422	HP	0.005**
	Gammaproteobacteria	*Enterobacteriales*				5.023	DP	0.036*
	Alphaproteobacteria	*Rhodobacterales*	*Rhodobacteraceae*			2.422	HP	0.005**
Proteobacteria	*Betaproteobacteria*	*Burkholderiales*	*Comamonadaceae*			2.471	HP	0.005**
	Gammaproteobacteria	*Enterobacteriales*	*Enterobacteriaceae*			4.305	DP	0.036*
	Gammaproteobacteria	*Xanthomonadales*	*Xanthomonadaceae*			1.774	HP	0.005**
	Betaproteobacteria	*Burkholderiales*	*Comamonadaceae*	*Other*		1.639	HP	0.005**
	Betaproteobacteria	*Burkholderiales*	*Comamonadaceae*	*Delftia*		1.636	HP	0.005**
	Gammaproteobacteria	*Xanthomonadales*	*Xanthomonadaceae*	*Stenotrophomonas*		1.774	HP	0.005**

	Alphaproteobacteria	*Rhodobacterales*	*Rhodobacteraceae*	*Rhodobacter*		2.422	HP	0.005**
	Betaproteobacteria	*Burkholderiales*	*Comamonadaceae*	*Comamonas*		1.809	HP	0.005**
	Betaproteobacteria	*Burkholderiales*	*Comamonadaceae*	*Leptothrix*		1.936	HP	0.005**
	Gammaproteobacteria	*Enterobacteriales*	*Enterobacteriaceae*	*Other*		4.924	DP	0.036*
	Gammaproteobacteria	*Pseudomonadales*	*Pseudomonadaceae*	*Pseudomonas*	*viridiflava*	1.636	HP	0.005**
Actinobacteria	*Actinobacteria*	*Actinomycetales*	*Propionibacteriaceae*			1.685	HP	0.005**
	Actinobacteria	*Actinomycetales*	*Propionibacteriaceae*	*Propionibacterium*		1.685	HP	0.005**
	Actinobacteria	*Actinomycetales*	*Propionibacteriaceae*	*Propionibacterium*	*acnes*	1.685	HP	0.005**

h. AP vs. DP

Phylum	Class	Order	Family	Genus	Species	LDA Score	Riched group	p-value
Firmicutes	*Clostridia*	*Clostridiales*				4.657	AP	0.021*
	Clostridia	*Clostridiales*	*Veillonellaceae*			4.846	DP	0.044*
	Erysipelotrichi	*Erysipelotrichales*	*Erysipelotrichaceae*			3.390	DP	0.041*
	Clostridia	*Clostridiales*	*Ruminococcaceae*	*Other*		4.286	AP	0.036*
	Bacilli	*Bacillales*	*Bacillales*	*Other*		2.070	AP	0.035*
	Clostridia	*Clostridiales*	*Clostridiaceae*	*SMB53*		3.997	AP	0.004**
	Clostridia	*Clostridiales*	*Veillonellaceae*	*Phascolarctobacterium*		3.015	AP	0.038*
	Clostridia	*Clostridiales*	*Veillonellaceae*	*Veillonella*	*Other*	4.826	DP	0.040*
	Bacilli	*Lactobacillales*	*Lactobacillaceae*	*Lactobacillus*	*mucosae*	3.814	DP	0.028*
	Bacilli	*Lactobacillales*	*Lactobacillaceae*	*Lactobacillus*	*vaginalis*	4.828	DP	0.012*
Bacteroidetes	*Bacteroidia*	*Bacteroidales*				4.038	AP	0.042*
	Bacteroidia	*Bacteroidales*	*Other*			3.615	AP	0.021*
	Bacteroidia	*Bacteroidales*	*Paraprevotellaceae*			2.086	AP	0.041*

	Bacteroidia	*Bacteroidales*	*Prevotellaceae*	*Prevotella*		4.084	AP	0.021*
	Bacteroidia	*Bacteroidales*	*Odoribacteraceae*	*Odoribacter*		3.003	AP	0.020*
	Bacteroidia	*Bacteroidales*	*Porphyromonadaceae*	*Porphyromonas*		3.284	DP	0.010*
	Bacteroidia	*Bacteroidales*	*Prevotellaceae*	*Prevotella*	*copri*	3.136	AP	0.033*
	Bacteroidia	*Bacteroidales*	*Porphyromonadaceae*	*Parabacteroides*	*Other*	2.280	AP	0.047*
	Bacteroidia	*Bacteroidales*	*Prevotellaceae*	*Prevotella*	*Other*	3.249	AP	0.020*
	Betaproteobacteria					4.424	AP	0.008**
Proteobacteria	*Betaproteobacteria*	*Burkholderiales*				4.421	AP	0.007**
	Gammaproteobacteria	*Pasteurellales*	*Pasteurellaceae*	*Other*		3.253	DP	0.008**
	Mollicutes					2.863	AP	0.032*
Tenericutes	*Mollicutes*	*RF39*				2.569	AP	0.007**
	Mollicutes	*Anaeroplasmatales*				2.536	AP	0.044*

Figure S1

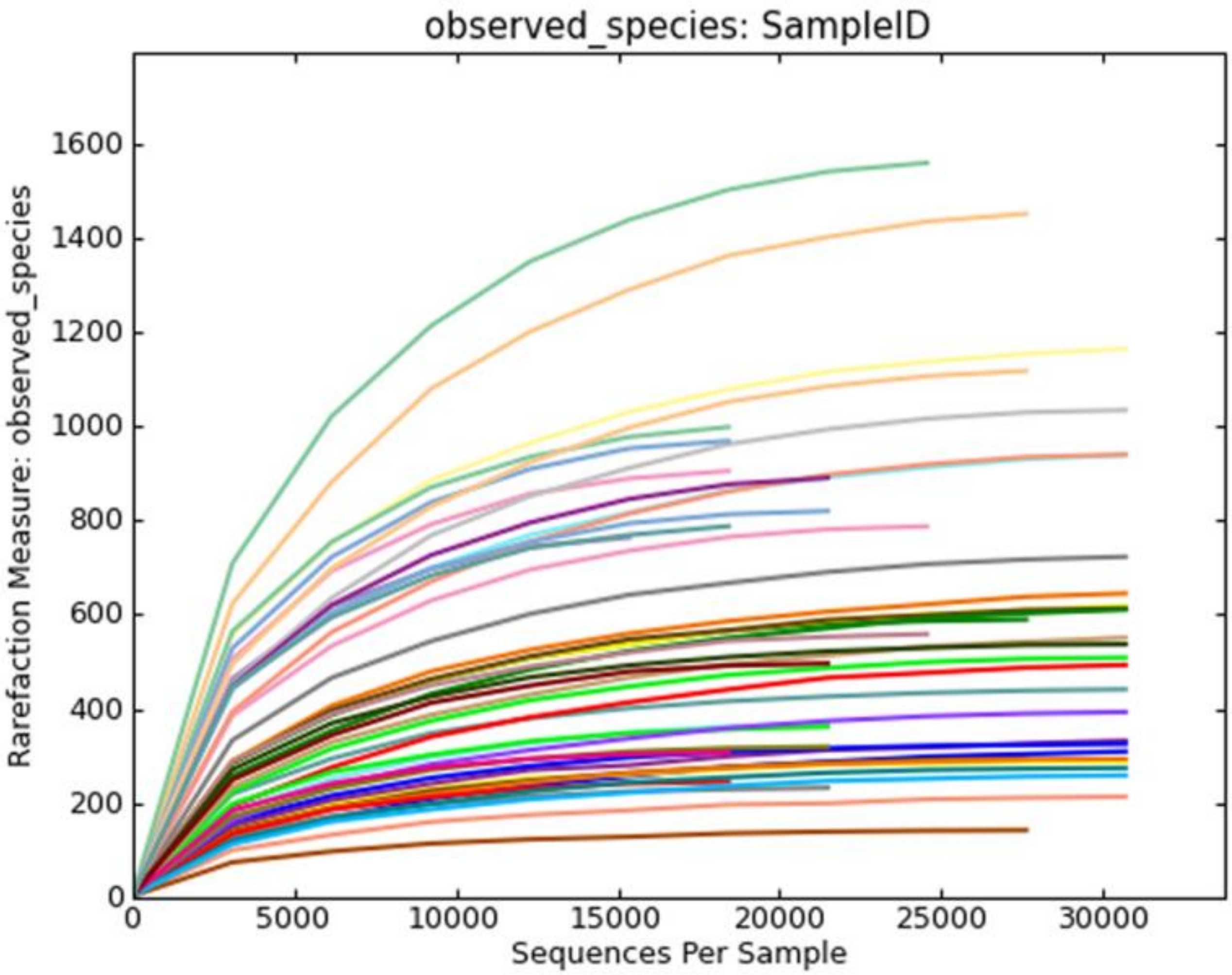

observed_species: SampleID
Rarefaction Measure: observed_species
Sequences Per Sample
0
200
400
600
800
1000
1200
1400
1600
5000
10000
15000
20000
25000
30000

Figure S2

(A)

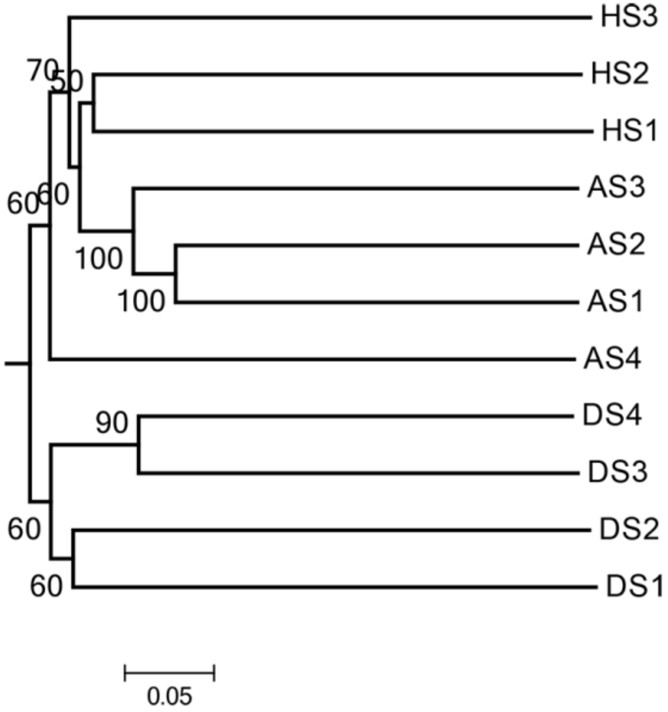
HS3
HS2
HS1
AS3
AS2
AS1
AS4
DS4
DS3
DS2
DS1
70
50
60
60
100
100
90
60
60
0.05

(B)

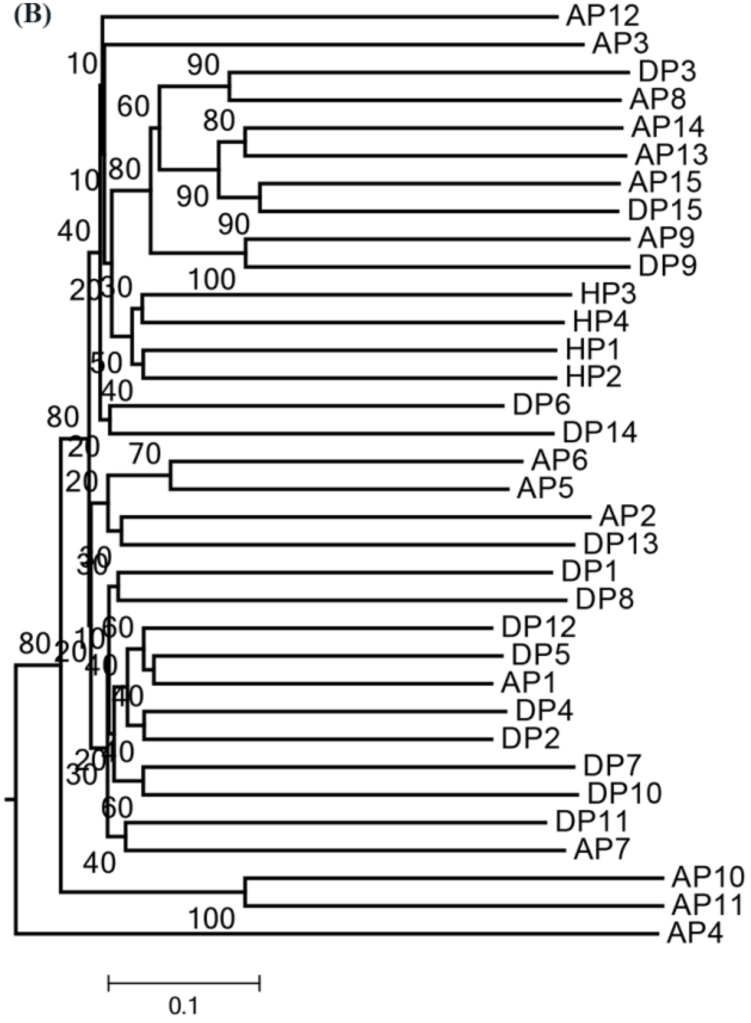
AP12
AP3
DP3
AP8
AP14
AP13
AP15
DP15
AP9
DP9
HP3
HP4
HP1
HP2
DP6
DP14
AP6
AP5
AP2
DP13
DP1
DP8
DP12
DP5
AP1
DP4
DP2
DP7
DP10
DP11
AP7
AP10
AP11
AP4
0.1

Figure S3

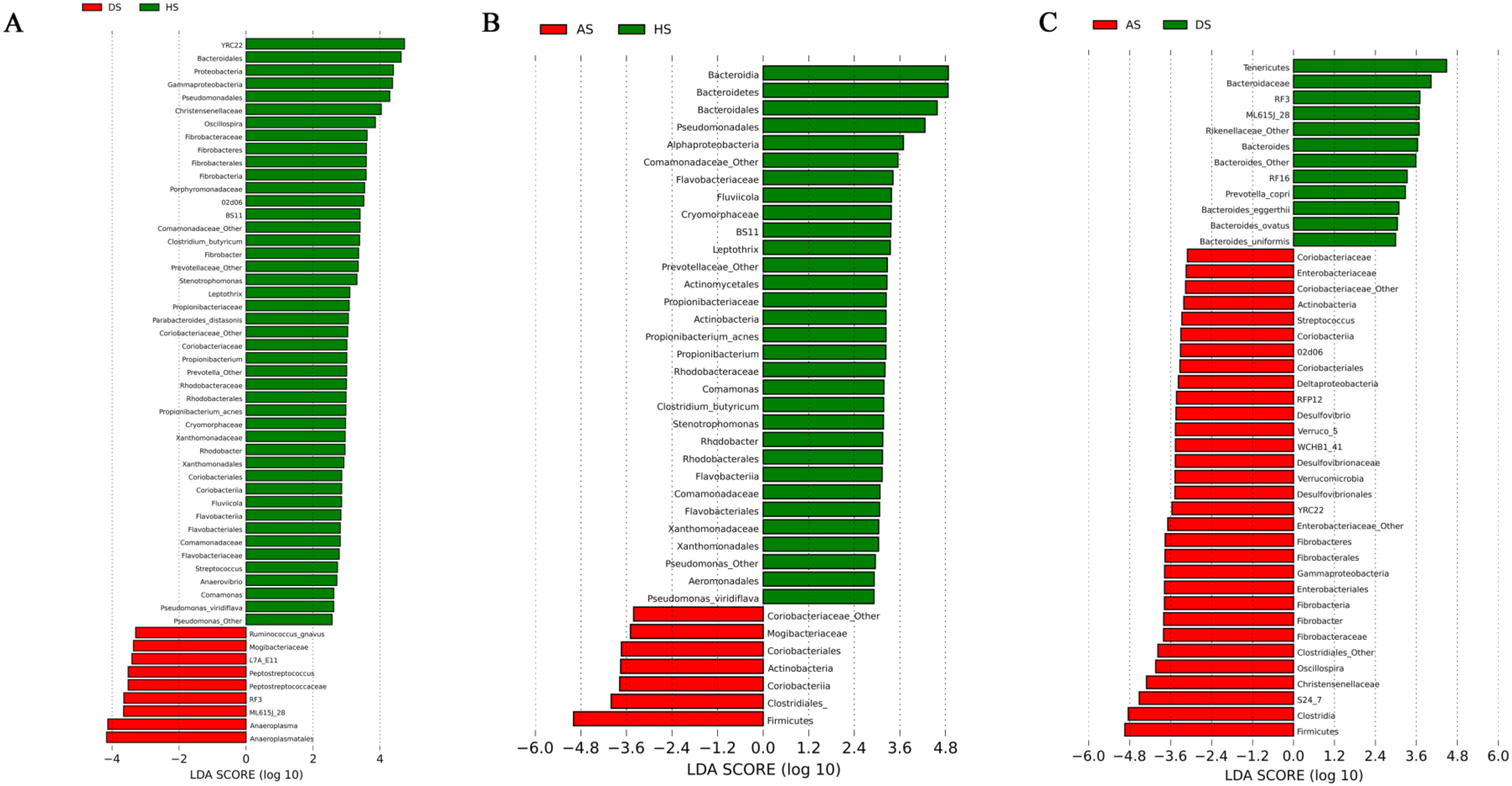

A
DS
HS
YRC22
Bacteroidales
Proteobacteria
Gammaproteobacteria
Pseudomonadales
Christensenellaceae
Oscillospira
Fibrobacteraceae
Fibrobacteres
Fibrobacterales
Fibrobacteria
Porphyromonadaceae
02d06
BS11
Comamonadaceae_Other
Clostridium_butyricum
Fibrobacter
Prevotellaceae_Other
Stenotrophomonas
Leptothrix
Propionibacteriaceae
Parabacteroides_distasonis
Coriobacteriaceae_Other
Coriobacteriaceae
Propionibacterium
Prevotella_Other
Rhodobacteraceae
Rhodobacterales
Propionibacterium_acnes
Cryomorphaceae
Xanthomonadaceae
Rhodobacter
Xanthomonadales
Coriobacteriales
Coriobacteriia
Fluviicola
Flavobacteriia
Flavobacteriales
Comamonadaceae
Flavobacteriaceae
Streptococcus
Anaerovibrio
Comamonas
Pseudomonas_viridiflava
Pseudomonas_Other
Ruminococcus_gnavus
Mogibacteriaceae
L7A_E11
Peptostreptococcus
Peptostreptococcaceae
RF3
ML615J_28
Anaeroplasma
Anaeroplasmatales
−4 −2 0 2 4
LDA SCORE (log 10)
B
AS
HS
Bacteroidia
Bacteroidetes
Bacteroidales
Pseudomonadales
Alphaproteobacteria
Comamonadaceae_Other
Flavobacteriaceae
Fluviicola
Cryomorphaceae
BS11
Leptothrix
Prevotellaceae_Other
Actinomycetales
Propionibacteriaceae
Actinobacteria
Propionibacterium_acnes
Propionibacterium
Rhodobacteraceae
Comamonas
Clostridium_butyricum
Stenotrophomonas
Rhodobacter
Rhodobacterales
Flavobacteriia
Comamonadaceae
Flavobacteriales
Xanthomonadaceae
Xanthomonadales
Pseudomonas_Other
Aeromonadales
Pseudomonas_viridiflava
Coriobacteriaceae_Other
Mogibacteriaceae
Coriobacteriales
Actinobacteria
Coriobacteriia
Clostridiales_
Firmicutes
−6.0 −4.8 −3.6 −2.4 −1.2 0.0 1.2 2.4 3.6 4.8
LDA SCORE (log 10)
C
AS
DS
Tenericutes
Bacteroidaceae
RF3
ML615J_28
Rikenellaceae_Other
Bacteroides
Bacteroides_Other
RF16
Prevotella_copri
Bacteroides_eggerthii
Bacteroides_ovatus
Bacteroides_uniformis
Coriobacteriaceae
Enterobacteriaceae
Coriobacteriaceae_Other
Actinobacteria
Streptococcus
Coriobacteriia
02d06
Coriobacteriales
Deltaproteobacteria
RFP12
Desulfovibrio
Verruco_5
WCHB1_41
Desulfovibrionaceae
Verrucomicrobia
Desulfovibrionales
YRC22
Enterobacteriaceae_Other
Fibrobacteres
Fibrobacterales
Gammaproteobacteria
Enterobacteriales
Fibrobacteria
Fibrobacter
Fibrobacteraceae
Clostridiales_Other
Oscillospira
Christensenellaceae
S24_7
Clostridia
Firmicutes
−6.0 −4.8 −3.6 −2.4 −1.2 0.0 1.2 2.4 3.6 4.8 6.0
LDA SCORE (log 10)

Figure S4

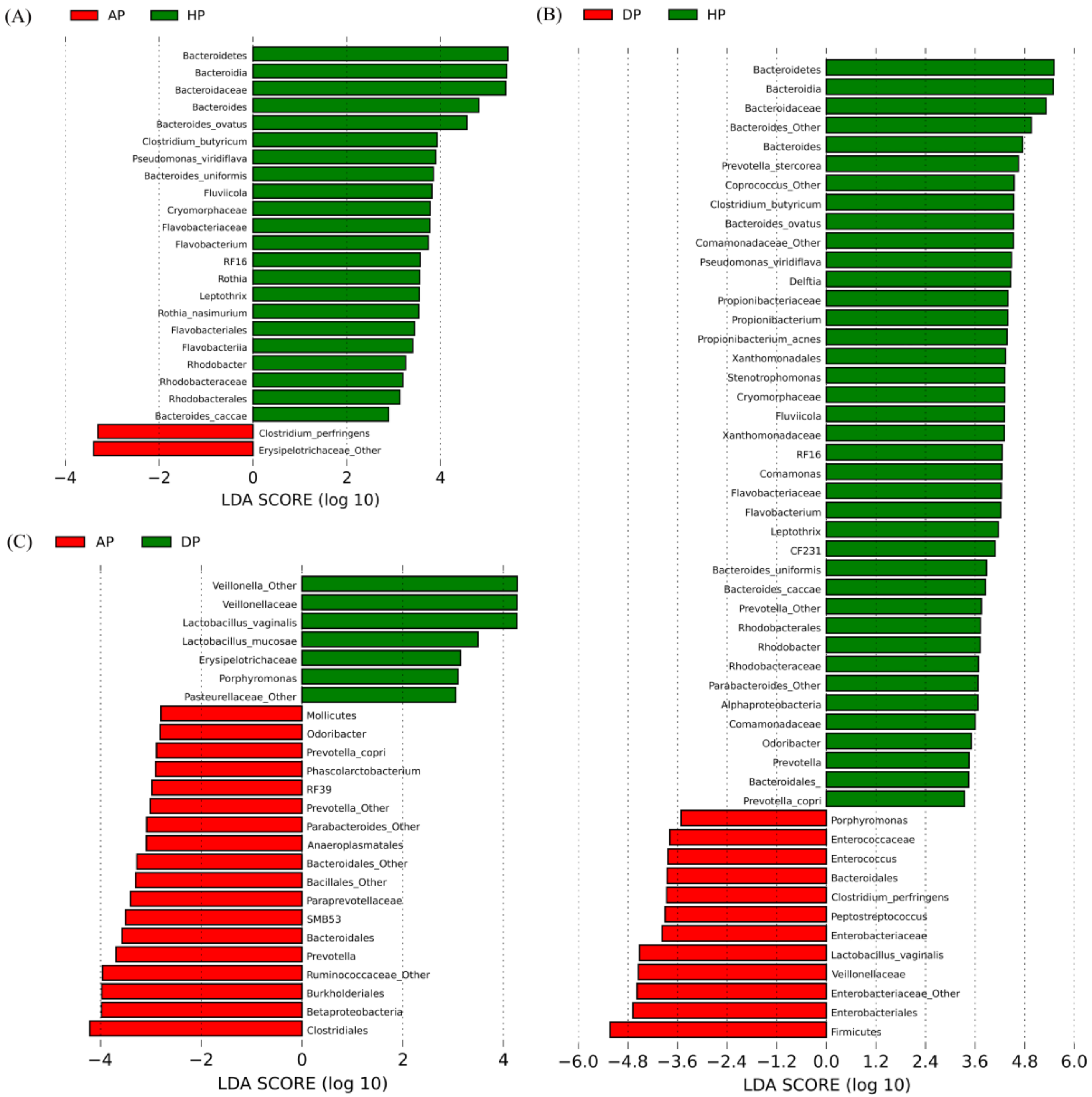
(A)
AP
HP
Bacteroidetes
Bacteroidia
Bacteroidaceae
Bacteroides
Bacteroides_ovatus
Clostridium_butyricum
Pseudomonas_viridiflava
Bacteroides_uniformis
Fluviicola
Cryomorphaceae
Flavobacteriaceae
Flavobacterium
RF16
Rothia
Leptothrix
Rothia_nasimurium
Flavobacteriales
Flavobacteriia
Rhodobacter
Rhodobacteraceae
Rhodobacterales
Bacteroides_caccae
Clostridium_perfringens
Erysipelotrichaceae_Other
−4
−2
0
2
4
LDA SCORE (log 10)
(B)
DP
HP
Bacteroidetes
Bacteroidia
Bacteroidaceae
Bacteroides_Other
Bacteroides
Prevotella_stercorea
Coprococcus_Other
Clostridium_butyricum
Bacteroides_ovatus
Comamonadaceae_Other
Pseudomonas_viridiflava
Delftia
Propionibacteriaceae
Propionibacterium
Propionibacterium_acnes
Xanthomonadales
Stenotrophomonas
Cryomorphaceae
Fluviicola
Xanthomonadaceae
RF16
Comamonas
Flavobacteriaceae
Flavobacterium
Leptothrix
CF231
Bacteroides_uniformis
Bacteroides_caccae
Prevotella_Other
Rhodobacterales
Rhodobacter
Rhodobacteraceae
Parabacteroides_Other
Alphaproteobacteria
Comamonadaceae
Odoribacter
Prevotella
Bacteroidales_
Prevotella_copri
Porphyromonas
Enterococcaceae
Enterococcus
Bacteroidales
Clostridium_perfringens
Peptostreptococcus
Enterobacteriaceae
Lactobacillus_vaginalis
Veillonellaceae
Enterobacteriaceae_Other
Enterobacteriales
Firmicutes
−6.0
−4.8
−3.6
−2.4
−1.2
0.0
1.2
2.4
3.6
4.8
6.0
LDA SCORE (log 10)
(C)
AP
DP
Veillonella_Other
Veillonellaceae
Lactobacillus_vaginalis
Lactobacillus_mucosae
Erysipelotrichaceae
Porphyromonas
Pasteurellaceae_Other
Mollicutes
Odoribacter
Prevotella_copri
Phascolarctobacterium
RF39
Prevotella_Other
Parabacteroides_Other
Anaeroplasmatales
Bacteroidales_Other
Bacillales_Other
Paraprevotellaceae
SMB53
Bacteroidales
Prevotella
Ruminococcaceae_Other
Burkholderiales
Betaproteobacteria
Clostridiales
−4
−2
0
2
4
LDA SCORE (log 10)
